# Comprehensive Characterization of *Toxoplasma* Acyl Coenzyme A-Binding Protein TgACBP2 and Its Critical Role in Parasite Cardiolipin Metabolism

**DOI:** 10.1128/mBio.01597-18

**Published:** 2018-10-23

**Authors:** Yong Fu, Xia Cui, Sai Fan, Jing Liu, Xiao Zhang, Yihan Wu, Qun Liu

**Affiliations:** a National Animal Protozoa Laboratory, College of Veterinary Medicine, China Agricultural University, Beijing, China; b Key Laboratory of Animal Epidemiology of the Ministry of Agriculture, College of Veterinary Medicine, China Agricultural University, Beijing, China; c Beijing Key Laboratory of Diagnostic and Traceability Technologies for Food Poisoning, Beijing Research Centre for Preventive Medicine, Beijing, China; d Centre for Disease Control and Prevention, Institute of Nutrition and Food Hygiene, Beijing, China; University of Pittsburgh

**Keywords:** *Toxoplasma gondii*, acyl-CoA-binding protein, cardiolipin metabolism, gene disruption, protein localization

## Abstract

Acyl coenzyme A (CoA)-binding protein (ACBP) can bind acyl-CoAs with high specificity and affinity, thus playing multiple roles in cellular functions. Mitochondria of the apicomplexan parasite Toxoplasma gondii have emerged as key organelles for lipid metabolism and signaling transduction. However, the rationale for how this parasite utilizes acyl-CoA-binding protein to regulate mitochondrial lipid metabolism remains unclear. Here, we show that an ankyrin repeat-containing protein, TgACBP2, is localized to mitochondria and displays active acyl-CoA-binding activities. Dephosphorylation of TgACBP2 is associated with relocation from the plasma membrane to the mitochondria under conditions of regulation of environmental [K^+^]. Under high [K^+^] conditions, loss of ACBP2 induced mitochondrial dysfunction and apoptosis-like cell death. Disruption of ACBP2 caused growth and virulence defects in the type II strain but not in type I parasites. Interestingly, mitochondrial association factor-1 (MAF1)-mediated host mitochondrial association (HMA) restored the growth ability of ACBP2-deficient type II parasites. Lipidomics analysis indicated that ACBP2 plays key roles in the cardiolipin metabolism of type II parasites and that MAF1 expression complemented the lipid metabolism defects of ACBP2-deficient type II parasites. In addition, disruption of ACBP2 caused attenuated virulence of Prugniuad (Pru) parasites for mice. Taking the results collectively, these data indicate that ACBP2 is critical for the growth and virulence of type II parasites and for the growth of type I parasites under high [K^+^] conditions.

## Abstract (precis)

Toxoplasma gondii is one of the most successful human parasites, infecting nearly one-third of the total world population. T. gondii tachyzoites residing within parasitophorous vacuoles (PVs) can acquire fatty acids both via salvage from host cells and via *de novo* synthesis pathways for membrane biogenesis. However, although fatty acid fluxes are known to exist in this parasite, how fatty acids flow through *Toxoplasma* lipid metabolic organelles, especially mitochondria, remains unknown. In this study, we demonstrated that *Toxoplasma* expresses an active ankyrin repeat containing protein TgACBP2 to coordinate cardiolipin metabolism. Specifically, HMA acquisition resulting from heterologous functional expression of MAF1 rescued growth and lipid metabolism defects in ACBP2-deficient type II parasites, manifesting the complementary role of host mitochondria in parasite cardiolipin metabolism. This work highlights the importance of TgACBP2 in parasite cardiolipin metabolism and provides evidence for metabolic association of host mitochondria with T. gondii.

## INTRODUCTION

As an obligate intracellular apicomplexan parasite, Toxoplasma gondii is widely recognized as an opportunistic pathogen and causes severe toxoplasmosis in pregnant women and immunocompromised individuals. The pathological manifestation of toxoplasmosis depends mainly on its robust lytic cycle, which requires abundant lipids for membrane biogenesis and signaling transduction. *Toxoplasma* harbors three independent pathways for fatty acid synthesis, namely, the apicoplast-localized FASII pathway, the fatty acid elongation pathway in the endoplasmic reticulum (ER), and the uncharacterized FASI pathway (1). The FASII pathway is required for the *de novo* synthesis of myristic acid (C14:0) and palmitic acid (C16:0) and plays essential roles in the growth and infectivity of this rapidly replicating parasite (2, 3). Apicoplast-synthesized fatty acids are further processed for elongation and desaturation by the ER-associated fatty acid elongation pathway (4). These fatty acids are used for lysophosphatidic acid (LPA) and phosphatidic acid (PA) precursor synthesis and then primarily serve as major backbones for the biosynthesis of numerous phospholipids, such as phosphatidylethanolamine (PE), phosphatidylcholine (PC), and phosphatidylinositol (PI) (5).

Long-chain acyl coenzyme A (LCACoA) molecules, specifically, phospholipids, triacylglycerol (TAG), cholesterol esters, and ceramide, play central roles in lipid metabolism as intermediates and are channeled to lipid metabolic pathways in various organelles by various transporters. Considering the amphipathicity of fatty acyl-CoA esters, membranes could be seriously damaged by acyl-CoAs acting as a detergent during movement (6). To avoid that effect, acyl-CoA-binding protein (ACBP), sterol carrier protein-2 (SCP2), and fatty acid-binding protein (FABPs) can all serve as trafficking machineries to target specific metabolic organelles where fatty acids are required (7–9). ACBP is the major carrier protein responsible for transporting acyl-CoA esters in cells and binds only middle-chain and long-chain fatty acyl-CoA esters and not free fatty acids, acylcarnitine, or cholesterol (7, 10). ACBP displays much higher affinity for acyl-CoA esters than FABP and SCP2 (7, 11). ACBP regulates enzymatic activity, vesicular trafficking, β-oxidation, and lipid metabolism by carrying long-chain fatty acyl-CoA esters and, more importantly, as an acyl-CoA pool to swiftly provide acyl chains for various metabolic pathways (12–15). Recent studies have demonstrated that six ACBP members in Arabidopsis thaliana play different roles in plant development and stress response to various environments (16). In addition, disruption of an ACBP homologue in wild-type yeast induces defects in sphingolipid synthesis and leads to aberrant membrane structures (17). Taking the results collectively, data have shown that ACBP has multiple cellular functions in yeast, plants, and animals and may also play significant roles in parasites.

While no candidate genes of FABP could be identified in the *Toxoplasma* genome (http://toxodb.org/toxo/), the *Toxoplasma* genome appears to encode two homologues of ACBP (TgACBP1 and TgACBP2). The third acyl-CoA transporter, SCP2, is expressed in T. gondii tachyzoites as an ancestral di/bifunctional protein containing two sterol carrier protein-2 domains (hence the name “SCP2”) and plays significant roles in lipid uptake and trafficking in *Toxoplasma* (18). Interestingly, another apicomplexan parasite, *Cryptosporidium*, harbors a parasitophorous vacuole membrane (PVM)-associated acyl-CoA-binding protein (CpACBP1), which is possibly involved in lipid remodeling and fatty acid transport across the PVM (19). However, roles of acyl-CoA-binding proteins in *Toxoplasma* are still unknown.

Here, we show that mitochondrion-localized ACBP2 plays key roles in the anti-apoptosis-like cell death pathway under high [K^+^] conditions. Disruption of ACBP2 caused growth defects in type II parasites rather than type I parasites and attenuated the abundance of cardiolipin (CL). Interestingly, mitochondrial association factor-1 (MAF1)-mediated host mitochondrial association (HMA) successfully rescued the growth and lipid metabolism defects of ACBP2-deficient type II parasites, demonstrating the complementary role of host mitochondria in lipid metabolism of *Toxoplasma*. These results reveal the multiple roles of ACBP2 in stress response and lipid metabolism in *Toxoplasma*.

## RESULTS

### TgACBP2 displays active acyl-CoA-binding activities *in vitro* and *in vivo*.

**FIG 1:**
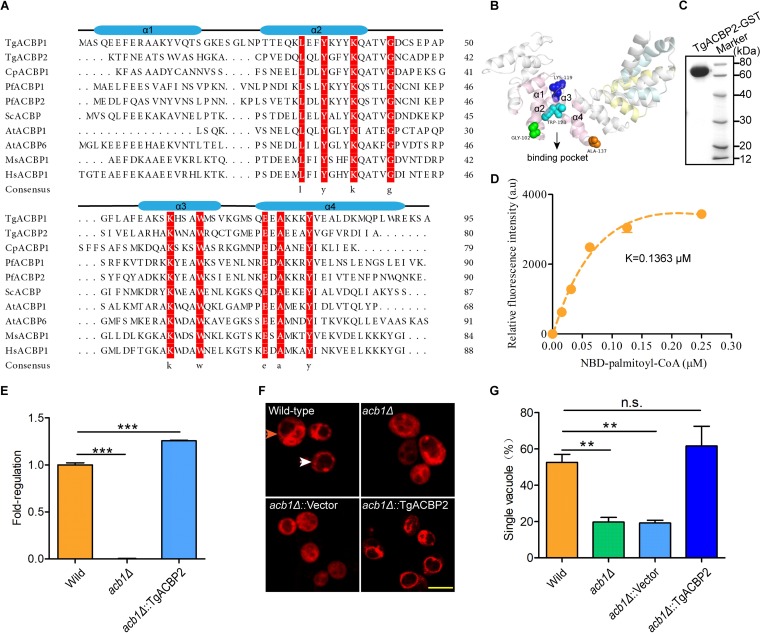
Characterization of structures and binding activities of TgACBP2. (A) Protein sequence alignment of ACBPs from T. gondii, Cryptosporidium parvum, P. falciparum, S. cerevisiae, A. thaliana, Mus musculus, and Homo sapiens was analyzed using MEGA7. “Consensus” indicates highly conserved amino acid residues. The secondary structures of ACBPs with four α-helix bundles labeled by blue cylinders are indicated above the sequence alignment. (B) 3D structure of TgACBP2 was predicted by homologous modeling using Swiss model. Conserved amino acid residues (colorful balls) of the binding pocket (arrow) are shown. α-Helices 1 to 4 of the acyl-CoA-binding domain of TgACBP2 are indicated, while closely aligned α-helices of TgACBP2 are shown in different colors. (C) Recombinant TgACBP2 with GST tag was expressed in E. coli and purified followed by SDS-PAGE analysis. (D) The binding kinetics of recombinant TgACBP2 was determined by a fluorescence assay in the presence of various NBD-C16:0-CoA concentrations. The bars represent the standard errors of the means (SEM) of results from two independent experiments, and “a.u.” represents “arbitrary units.” (E to G) Complementation of the *acb1*Δ yeast vacuole phenotype by heterologous expression of TgACBP2. TgACBP2 was transfected into *acb1*Δ yeast (*acb1*Δ::TgACBP2). *acb1*Δ::empty vector yeasts were used as a control. Reverse transcription-PCR (RT-PCR) showed that the transcriptional level of TgACBP2 in strain *acb1*Δ::TgACBP2 is higher than that of the original ACBP of wild-type yeast (E). Yeasts were stained with the fluorescent dye FM1-43 and observed by confocal microscopy. Yeasts with single- and multilobed vacuoles are shown by white and orange arrows, respectively (F). The numbers of yeasts with different vacuole phenotypes were counted for at least 100 yeasts (G). Data are presented as the means ± SEM of results from three independent experiments.

Bioinformatic analysis suggests that the *Toxoplasma* genome encodes an ACBP containing ankyrin repeat (http://toxodb.org/toxo/), T. gondii ACBP2 (TgACBP2 [TGGT1_234510]), which shares conserved residues in four α-helix bundles with other species (Fig. 1A). The three-dimensional (3D) structure of TgACBP2 shows spatial position of highly conserved residues, which are critical for the “binding pocket” of the acyl-CoA-binding domain constituted by α-helix bundles (Fig. 1B). All these structures correspond to the characteristics of canonical ACBP (20).

To investigate the binding activity of TgACBP2 *in vitro*, we expressed recombinant TgACBP2 with a C-terminal glutathione *S*-transferase (GST) tag in Escherichia coli (Fig. 1C). Subsequently, a binding assay was performed using the fluorescent substrate 4-nitrobenzo-2-oxa-1, 3-diazole-labeled C16:0-CoA (NBD-C16:0-CoA) to confirm the role of TgACBP2 in binding long-chain acyl-CoA ester. Nitrobenzoxadiazole (NBD) is nearly nonfluorescent in aqueous solution but can produce increased fluorescence in a polar environment such as in the binding pocket of an enzyme. The result demonstrated the binding constant of TgACBP2 for NBD-C16:0-CoA (K = 0.1363 μM) (Fig. 1D), which is comparable to the binding constant of CpACBP1 for NBD-C16:0-CoA. In addition, wild-type Saccharomyces cerevisiae vacuoles have equal proportions of single- and multilobed vacuoles, while the disruption of ACBP in yeasts causes vacuole disintegration and an increasing number of yeasts with multilobed vacuoles (17). To further investigate the binding activity of TgACBP2 *in vivo*, we expressed TgACBP2 in S. cerevisiae *acb1*Δ mutant (Fig. 1E) and determined the proportions of single-lobed vacuoles in different yeasts. Data showed that *acb1*Δ yeasts expressing TgACBP2 reversed the multilobed vacuole phenotype and balanced the number of cells with single- and multilobed vacuoles (Fig. 1F). Taking the results collectively, these results demonstrated that TgACBP2 is active *in vitro* and *in vivo*.

### TgACBP2 is localized to the mitochondria in intracellular parasites and to the periphery in extracellular parasites.

**FIG 2:**
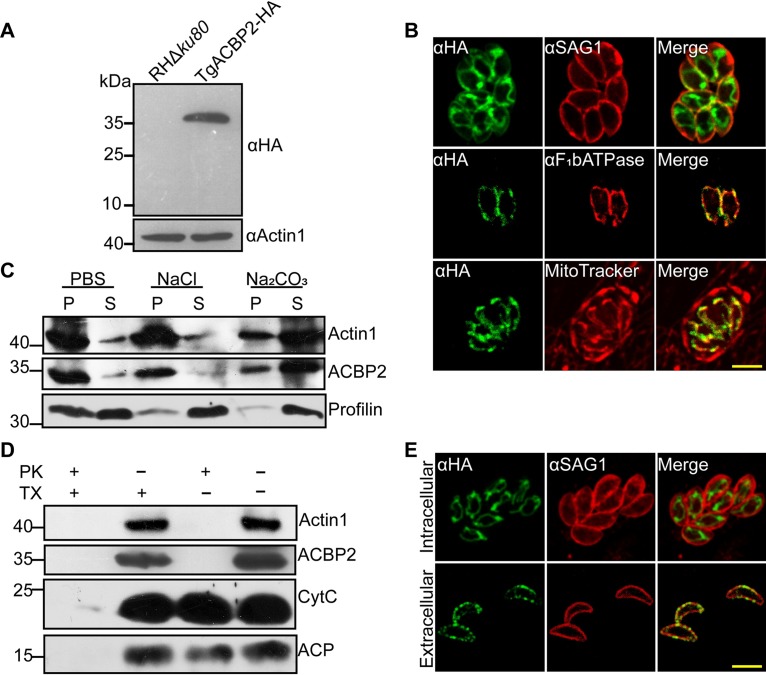
Localization of TgACBP2 in extracellular and intracellular parasites. (A) Western blot analysis of TgACBP2-HA produced by endogenous C-terminal tagging. Parasite lysates were probed with anti-HA (upper panel); Actin1 served as the loading control (lower panel). (B) Localization of TgACBP2 was further determined by IFA using two different mitochondrial markers, F_1_bATPase and MitoTrackerRed CMXRos. Anti-HA was used to label TgACBP2, and SAG1 was used as a parasite membrane marker. Bars = 5 μm. (C) The solubility of TgACBP2 was investigated by fractionation experiments after the ultrasonication of tachyzoites in PBS, NaCl, and Na_2_CO_3_ solutions. The distribution of TgACBP2 fractions was measured by Western blotting using mouse anti-TgACBP2 antibody; Actin1 and Profilin were used as controls for membrane-associated and soluble proteins, respectively. (D) A proteinase K protection assay was used to determine the accessibility of ACBP2 to proteinase K in the presence or absence of the detergent Triton X-100 (TX). After permeabilization by TX, PK could easily pass through organelle membranes to stroma and digest stoma proteins nonspecifically. For ACBP2, even without TX, PK was also able to degrade ACBP2, further indicating that ACBP2 is localized on the membrane of mitochondria. Actin1 served as a marker of cytosolic proteins, while ACP and cytochrome *c* (CytC) served as markers of apicoplast stroma and inner mitochondria, respectively. Mouse anti-ACBP2 antibody was used to detect the expression of ACBP2. (E) The subcellular localization of ACBP2 was determined in both intracellular and extracellular tachyzoites endogenously tagged with TgACBP2-HA. Anti-HA was used to detect the distribution of ACBP2, and SAG1 was used as a membrane marker. ACBP2 was localized to mitochondria in intracellular tachyzoites and yet translocated to the plasma membrane in extracellular tachyzoites. Bars = 5 μm.

To reveal the localization of TgACBP2, we constructed hemagglutinin (HA) epitope-tagged TgACBP2 by single homologous recombination in RHΔ*ku80* parasites. Western blotting verified the expected molecular mass of ∼36 kDa for TgACBP2-HA (Fig. 2A). Immunofluorescence assays (IFAs) showed that TgACBP2 was localized to mitochondria using two mitochondrial markers, MitoTracker and F_1_bATPase, while MitoTracker could also stain host mitochondria (Fig. 2B). To study the solubility of TgACBP2, fractionation experiments were performed. Western blotting revealed that TgACBP2 is partially soluble in phosphate-buffered saline (PBS) and high concentrations of salt, while its solubility was significantly increased in carbonate buffer, indicative of its association with the mitochondrial membrane (Fig. 2C). Next, we investigated the membrane topology of TgACBP2. A proteinase K (PK) protection assay was completed, with apicoplast matrix protein ACP, cytochrome *c* (CytC), and Actin1 as controls. ACP and CytC were both fully protected from PK digestion without Triton X-100 permeabilization (Fig. 2D), indicative of the presence of proteins inside the organelles, while TgACBP2 and TgActin1 were both digested by PK even without the addition of detergent (Fig. 2D), suggesting that TgACBP2 may be localized to the mitochondrial membrane and may face the cytoplasm. Next, we determined the localization of TgACBP2 in intracellular and extracellular tachyzoites. TgACBP2 of intracellular tachyzoites was found to be localized to mitochondria, while TgACBP2 was relocated to the pellicles of extracellular tachyzoites (Fig. 2E). These data reveal different localizations of TgACBP2 in extracellular and intracellular parasites.

### Regulation of TgACBP2 redistribution and dephosphorylation relies on environmental [K^+^].

**FIG 3:**
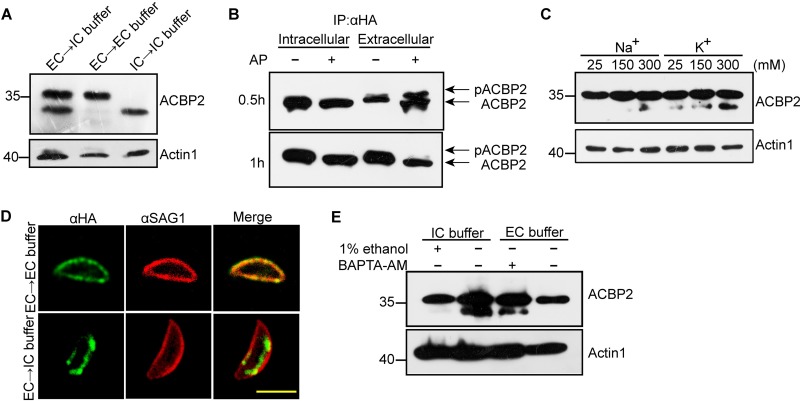
Environmental [K^+^]-induced dephosphorylation of ACBP2 is associated with its translocation. (A) Western blot analysis of ACBP2 expression in extracellular and intracellular tachyzoites. Intracellular and extracellular tachyzoites were harvested and incubated in IC buffer and EC buffer for 2 h, respectively. Additionally, extracellular tachyzoites were incubated in IC buffer for 2 h. Two forms of ACBP2 were present in extracellular and intracellular tachyzoites. Extracellular tachyzoites underwent stimulation by IC buffer and produced a smaller band consistent with the size of intracellular ACBP2. Mouse anti-ACBP2 antibody was used to detect the expression of ACBP2. Actin1 was used as the loading control. EC, extracellular tachyzoites; IC, intracellular tachyzoites. (B) Alkaline phosphatase (AP) assay for the phosphorylation of TgACBP2. Endogenous TgACBP2 immunoprecipitated (IP) from both intracellular and extracellular TgACBP2-HA parasites using antibodies against HA was incubated with or without AP for 0.5 h and 1 h; ACBP2 from extracellular tachyzoites began to be dephosphorylated (upper panel) and was completely dephosphorylated after 1 h (lower panel). “pACBP2” represents “phosphorylated ACBP2.” Mouse anti-ACBP2 antibody was used to detect the expression of ACBP2. (C) Extracellular TgACBP2-HA tachyzoites incubated with 25 mM, 125 mM, and 300 mM KCl and NaCl buffers for 2 h were analyzed by Western blotting using antibodies against HA. [K^+^] induced ACBP2 dephosphorylation in a concentration-dependent manner. Anti-HA was used to detect the expression of ACBP2. Actin1 was used as the loading control. (D) Extracellular TgACBP2-HA parasites were harvested and incubated with IC buffer for 2 h. IFA showed that IC buffer induced the translocation of TgACBP2. Anti-HA was used to detect the localization of ACBP2. Bar, 5 μm. (E) The association of [Ca^2+^] with TgACBP2 dephosphorylation was assessed using 1% ethanol and Ca^2+^ chelator BAPTA-AM. Extracellular TgACBP2-HA parasites were harvested and incubated in EC buffer and IC buffer. After the addition of 1% ethanol to the IC buffer, extracellular parasites in IC buffer were not dephosphorylated, while treatment of extracellular parasites with BAPTA-AM in EC buffer induced ACBP2 dephosphorylation. Anti-HA was used to detect the expression of ACBP2. Actin1 was used as the loading control.

To compare the levels of expression of TgACBP2 in intracellular and extracellular parasites, we scraped T. gondii-infected human foreskin fibroblasts (HFFs) into a high [K^+^] intracellular (IC) buffer and harvested completely egressed extracellular tachyzoites in extracellular (EC) buffer for 2 h of incubation. TgACBP2 of extracellular tachyzoites displayed a bigger band (∼36 kDa) than that of intracellular tachyzoites (Fig. 3A). When we transferred the extracellular tachyzoites into IC buffer, a smaller band consistant with the presence of intracellular TgACBP2 appeared in addition to the ∼36-kDa band (Fig. 3A). These results indicated that TgACBP2 may be posttranslationally modified in extracellular tachyzoites.

To investigate this possibility, TgACBP2s immunoprecipitated from both extracellular and intracellular ACBP2-HA tachyzoites were treated with or without a nonspecific alkaline phosphatase (AP) that could induce dephosphorylation by removing the phosphate groups (21). Extracellular TgACBP2 was found to be hydrolyzed after 0.5 h of AP treatment and fully dephosphorylated after 1 h of treatment (Fig. 3B), while intracellular TgACBP2 was nonreactive (Fig. 3B), demonstrating that TgACBP2 was phosphorylated in extracellular tachyzoites and unphosphorylated in intracellular tachyzoites. Interestingly, we used Phos-tag-based Western blotting to analyze the phosphorylation of ACBP2 in type II Prugniuad (Pru) parasites and found that ACBP2 could be phosphorylated in type I parasites whereas it was unphosphorylated in type II parasites (see Fig. S1A in the supplemental material), which was further verified in alkaline phosphatase assays of HA-tagged ACBP2 in type II parasites (Fig. S1B).

10.1128/mBio.01597-18.1 FIG S1 Phosphorylation analysis of ACBP2 in RHΔ*ku80* and Pru parasites. (A) RHΔ*ku80* and Pru parasites were harvested after natural egress. Phosphorylation of ACBP2 in RHΔ*ku80* and Pru parasites was analyzed by Phos-tag assay. The results showed the presence of a bigger band of phosphorylated ACBP2 than of unphosphorylated ACBP2, while ACBP2 in Pru did not display a band of phosphorylated ACBP2. (B) Alkaline phosphatase assay of ACBP2 in strain PruΔ*acbp2*/ACBP2 showed that neither intracellular nor extracellular Pru parasites could express phosphorylated ACBP2. Anti-HA was used to immunoprecipitate ACBP2 from PruΔ*acbp2*/ACBP2 parasites, while mouse anti-ACBP2 antibody was used to probe the expression of ACBP2. Download FIG S1, TIF file, 0.4 MB. Copyright © 2018 Fu et al. 2018 Fu et al. This content is distributed under the terms of the Creative Commons Attribution 4.0 International license.

As the IC buffer could induce TgACBP2 dephosphorylation in extracellular tachyzoites, we wanted to elucidate which ion mainly contributed to this induction. When extracellular tachyzoites were resuspended in KCl or NaCl buffers of various concentrations, increases in the environmental [K^+^] induced the dephosphorylation of TgACBP2 (Fig. 3C). However, no dephosphorylated TgACBP2 was detected after incubation with 25 and 150 mM NaCl buffers, indicating that the increased level of environmental [K^+^] rather than [Na^+^] results in the dephosphorylation of TgACBP2 in extracellular tachyzoites (Fig. 3C). These data suggest that [K^+^] plays key roles in the regulation of TgACBP2 dephosphorylation.

Next, we investigated whether the relocation of TgACBP2 in extracellular tachyzoites could be induced by high [K^+^]. Obviously, TgACBP2 of extracellular parasites in IC buffer was redistributed (Fig. 3D), while TgACBP2 was distributed around the periphery of extracellular parasites without induction of the high [K^+^] condition (Fig. 3D). Next, we determined whether the dephosphorylation of TgACBP2 was also dependent on Ca^2+^ signaling transduction, which could activate calcium-dependent protein kinases (CDPKs) (22). We resuspended extracellular tachyzoites in IC or EC buffer and then added 1% ethanol to stimulate the release of Ca^2+^ or 1 mM BAPTA-AM (Sigma) to chelate cytoplasmic Ca^2+^. The chelation of cytoplasmic Ca^2+^ resulted in TgACBP2 dephosphorylation in extracellular parasites, while the increased [Ca^2+^] induced by ethanol blocked the dephosphorylation of TgACBP2 (Fig. 3E). This indicates that dephosphorylation of TgACBP2 is regulated by [Ca^2+^].

These findings suggest that TgACBP2 of extracellular parasites could be dephosphorylated by the regulation of [K^+^] and [Ca^2+^] and relocated to mitochondria from the parasite periphery under high [K^+^] conditions.

### TgACBP2 is important for the efficient growth of parasites under high [K^+^] conditions.

**FIG 4:**
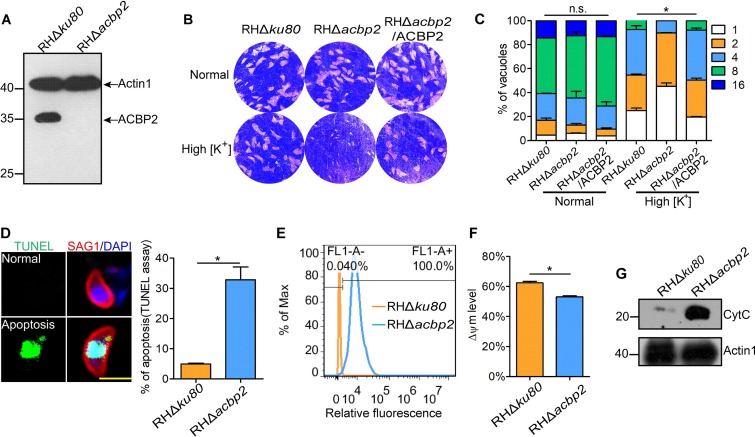
Loss of TgACBP2 caused mitochondrial dysfunction in parasites under high [K^+^] conditions. (A) TgACBP2 knockout was confirmed by Western blotting using mouse anti-ACBP2 antibody. Actin1 was used as the loading control. (B) Plaque assay showed that RHΔ*acbp2* tachyzoites treated under high [K^+^] (IC buffer) conditions displayed growth defects, while disruption of ACBP2 did not affect the growth phenotype of parasites under normal conditions. (C) After 2 h of incubation with IC buffer, RHΔ*ku80* and RHΔ*acbp2* tachyzoites were inoculated into HFFs and grown for 24 h in normal media. High [K^+^] stress impaired the intracellular replication of ACBP2-deficient tachyzoites. Data are represented as the means ± SEM of results from three assays. (D) Apoptosis of RHΔ*ku80* and RHΔ*acbp2* tachyzoites after 2 h of incubation with IC buffer was assessed by the TUNEL assay. The results showed that ACBP2 knockout resulted in an increased number of TUNEL-positive parasites (left panel). Results of IFA analyses of TUNEL-positive and TUNEL-negative parasites are shown in the right panel. Bar, 5 μm. Data are presented as the means ± SEM of results from three independent experiments. DAPI, 4′,6-diamidino-2-phenylindole. (E) Reactive oxygen species (ROS) levels were determined by FACS analysis using DCFH-DA. RHΔ*acbp2* parasites treated with IC buffer resulted in increased ROS levels. Max, maximum. (F) FACS measurement of ΔΨm in RHΔ*ku80* and RHΔ*acbp2* tachyzoite levels after 2 h of incubation with IC buffer was performed by DiOC6(3) staining. IC buffer treatment decreased the number of ΔΨm-positive parasites. The results are presented as the means ± SEM from four independent experiments. (G) Western blot analysis showed that substantial amounts of CytC were detected in the cytoplasmic extracts of RHΔ*acbp2* tachyzoites compared with the amounts in those of RHΔ*ku80* parasites after 2 h of incubation with IC buffer. Actin1 was used as the loading control.

To further investigate the functions of TgACBP2, we constructed a TgACBP2 deletion mutant by double homologous recombination in RHΔ*ku80* parasites (Fig. S2A). PCR analysis, fluorescence microscopy observations, and Western blotting confirmed the ACBP2 knockout (Fig. 4A; see also Fig. S2B and C). Complementation of TgACBP2 was also performed by insertion of the TgACBP2-HA open reading frame (ORF) into the *uprt* locus of RHΔ*acbp2* parasites, which was confirmed by Western blotting (Fig. S2E). A plaque assay showed no obvious differences in growth and proliferation between RHΔ*ku80* and RHΔ*acbp2* parasites (Fig. 4B). However, loss of TgACBP2 in RHΔ*ku80* parasites caused defective fitness in the competition assay (Fig. S2D).

10.1128/mBio.01597-18.2 FIG S2 Loss of ACBP2 in RHΔ*ku80* parasites induced weakened fitness. (A) Schematic representation of TgACBP2 knockout in RHΔ*ku80* parasites. (B) PCR analysis of TgACBP2 knockout in RHΔ*ku80* parasites. (C) Fluorescence microscopy showed RFP expression in RHΔ*acbp2* parasites. Bar, 10 μm. (D) A competition assay demonstrated that the loss of ACBP2 resulted in impaired parasite fitness compared with that of RHΔ*ku80* parasites after 6 passages. (E) Western blot analysis of ACBP2 complementation in ACBP2-deficient parasites. Download FIG S2, TIF file, 2.2 MB. Copyright © 2018 Fu et al. 2018 Fu et al. This content is distributed under the terms of the Creative Commons Attribution 4.0 International license.

As TgACBP2 in extracellular parasites could respond to high [K^+^], we evaluated the effects of high [K^+^] on RHΔ*acbp2* parasites. After 2 h of treatment in IC buffer, extracellular RHΔ*acbp2* parasites were inoculated into HFFs for the plaque and proliferation assays under normal conditions (23). The plaque assay showed that growth of the RHΔ*acbp2* parasites was obviously inhibited (Fig. 4B). Additionally, replication of the RHΔ*acbp2* parasites was repressed after high [K^+^] stress (Fig. 4C). Importantly, complementation of ACBP2 rescued the phenotype defects of RHΔ*acbp2* parasites after high [K^+^] stress (Fig. 4B and C). These data suggest that high [K^+^] stress may trigger cellular damage in RHΔ*acbp2* parasites.

T. gondii could respond quickly to chemotherapeutic agent-triggered cellular damage via the ancient apoptosis-like cell death pathway (24, 25). Therefore, we investigated the apoptosis of extracellular RHΔ*acbp2* parasites after high [K^+^] stress using the terminal deoxynucleotidyltransferase-mediated dUTP-biotin nick end labeling (TUNEL) assay. Approximately 30% of the RHΔ*acbp2* parasites were TUNEL positive after high [K^+^] stress (∼ 5% for RHΔ*ku80* parasites) (Fig. 4D), indicative of a significantly increased apoptosis rate.

Mitochondria play key roles in the apoptotic pathway, and mitochondrial dysfunction could trigger the apoptosis-like cell death pathway in T. gondii and Plasmodium falciparum (24, 26). Thus, we evaluated changes in levels of reactive oxygen species (ROS) and mitochondrial membrane potential (ΔΨm) in parasites after high [K^+^] stress. Fluorescence-activated cell sorter (FACS) measurement of the fluorescence of parasites stained with 2',7'-dichlorodihydrofluorescein diacetate (DCFH-DA) showed that RHΔ*acbp2* parasites treated with IC buffer exhibited increased fluorescence (Fig. 4E), reflecting increased ROS levels in the mitochondria of “stressed parasites.” Following treatment with IC buffer, a reduction in the ΔΨm of RHΔ*acbp2* parasites was observed compared with that of parental parasites using the DiOC6(3) (3,3'-dihexyloxacarbocyanine iodide) staining assay (Fig. 4F). Cytochrome *c* release from mitochondria is reportedly closely related to the mitochondrion-mediated apoptotic pathway (27). Western blot analysis of “stressed parasites” showed that larger amounts of cytochrome c were detected in the cytosolic extracts of RHΔ*acbp2* parasites compared to those of parental tachyzoites (Fig. 4G), indicating that loss of TgACBP2 induced cytochrome *c* release from mitochondria of parasites under conditions of high [K^+^] stress.

Together, these data demonstrate that the loss of TgACBP2 results in mitochondrial dysfunction in parasites under conditions of high [K^+^] stress and that TgACBP2 plays a role in preventing the apoptosis-like pathway in parasites, most likely by maintaining mitochondrial homeostasis.

### TgACBP2 is associated with cardiolipin metabolism in Pru tachyzoites.

**FIG 5:**
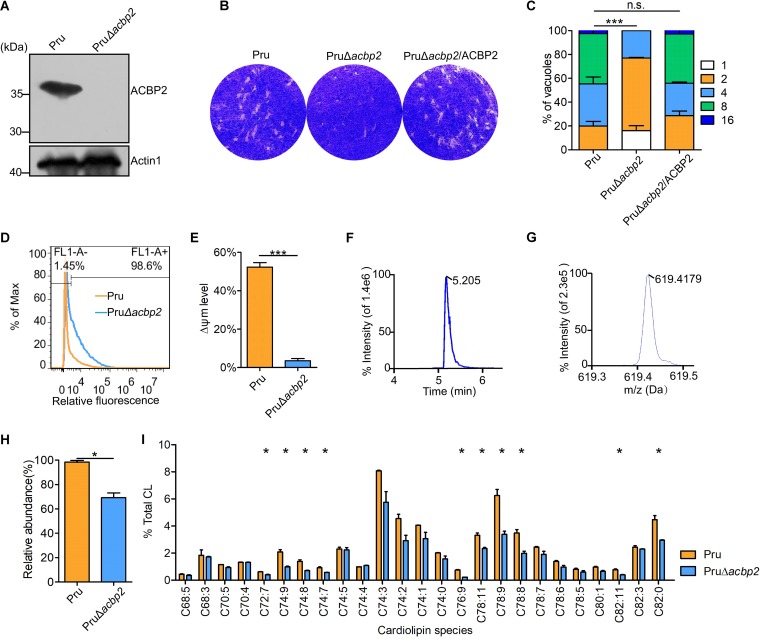
Loss of TgACBP2 in Pru tachyzoites caused defects in cardiolipin metabolism. (A) Western blotting confirmed disruption of TgACBP2 in Pru parasites using mouse anti-ACBP2 antibody. Actin1 was used as the loading control. The numbers of PruΔ*acbp2* tachyzoites represent different monoclones. (B) A plaque assay was performed by inoculating HFFs with 100 Pru and PruΔ*acbp2* tachyzoites for 12 days. ACBP2 knockout in the Pru strain resulted in impaired growth. (C) A proliferation assay showed that loss of ACBP2 in the Pru strain induced replication defects. Data are presented as the means ± SEM of results from three independent experiments. (D) ROS levels in Pru and PruΔ*acbp2* tachyzoites were measured by FACS analysis using the specific dye DCFH-DA. Loss of ACBP2 in the Pru strain increased the fluorescence intensity. (E) ΔΨm levels were determined in Pru and PruΔ*acbp2* tachyzoites by DiOC6(3) staining. FACS results showed that the PruΔ*acbp2* tachyzoites displayed significantly decreased numbers of ΔΨm-positive parasites. The error bars represent the standard errors of the means of results from three independent experiments. (F and G) Extracted ion chromatogram (F) and mass spectrum (G) of cardiolipin standard C56:0 are shown in HPLC/HRMS results. (H and I) Parental or PruΔ*acbp2* parasites were cultivated in HFFs and harvested. Cardiolipins were extracted from the parasites and analyzed by HPLC/HRMS. CL overall abundance analysis showed that loss of ACBP2 caused decreased overall abundances of CL in Pru (H). The relative abundances of 25 cardiolipin species are shown (I). Disruption of ACBP2 globally led to reduced intensities of the cardiolipin species. Data are normalized to the intensity of C56:0, which served as an internal standard. Data are presented as the means ± SEM of results from two independent experiments.

To further study the functions of TgACBP2, Pru tachyzoites lacking TgACBP2 were generated by homologous recombination. Positive monoclones were selected by PCR (Fig. S3A), and Western blot analysis confirmed the knockout and complementation of ACBP2 in Pru parasites (Fig. 5A; see also Fig. S3B). The growth rate of PruΔ*acbp2* parasites was significantly inhibited in the plaque assay (Fig. 5B) and was closely associated with impaired intracellular replication (Fig. 5C). However, neither apicoplast segregation nor inner membrane complex (IMC) formation was impaired following the disruption of ACBP2 in Pru tachyzoites (Fig. S3C and D).

10.1128/mBio.01597-18.3 FIG S3 TgACBP2 knockout in Pru parasites did not impair apicoplast segregation, inner membrane complex (IMC) formation, or mitochondrial biogenesis. (A) PCR identification of monoclones for the disruption of TgACBP2 in Pru parasites. (B) Western blotting showed that ACBP2-deficient parasites were complemented by the expression of HA-tagged ACBP2. (C to E) Loss of TgACBP2 in Pru tachyzoites did not affect apicoplast segregation (C), IMC formation (D), or mitochondrial biogenesis (E). ACP was used as a parasite apicoplast marker, while IMC1 and mitoTracker were used as cytoplasmic division and mitochondrial biogenesis markers, respectively. Bar, 5 μm. Download FIG S3, TIF file, 2.8 MB. Copyright © 2018 Fu et al. 2018 Fu et al. This content is distributed under the terms of the Creative Commons Attribution 4.0 International license.

Considering the localization of ACBP2 in intracellular tachyzoites, we hypothesized that the disruption of ACBP2 might cause mitochondrial dysfunction in Pru tachyzoites. While loss of TgACBP2 had no effects on mitochondrial morphology (Fig. S3E), FACS results showed markedly reduced Δψm (Fig. 5D) and, subsequently, elevated ROS levels in PruΔ*acbp2* parasites (Fig. 5E), demonstrating critical roles of ACBP2 in maintaining mitochondrial health in Pru parasites.

ACBP2 can bind long-chain fatty acyl-CoAs, which could be metabolized in mitochondria by two major pathways: the fatty acid oxidation and cardiolipin synthesis pathways. Previous research showed no obvious evidence for the presence of the fatty acid oxidation pathway in mitochondria (28). Therefore, we hypothesized that ACBP2 plays some roles in cardiolipin metabolism in *Toxoplasma* mitochondria. We established a method for detecting cardiolipin standard C56:0 using high-performance liquid chromatography (HPLC) coupled to high-resolution mass spectrometry (HRMS) (Fig. 5F and G). In Pru tachyzoites, we totally detected 25 kinds of cardiolipins (see Table S2 in the supplemental material) which were significantly different from cardiolipins of human cells. Loss of ACBP2 globally reduced cardiolipin abundance and especially decreased the relative abundances of C72:7, C74:9, C74:8, C74:7, C76:9, C78:11, C78:9, C78:8, C82:11, and C82:0 (Fig. 5H). Taking the data together, ACBP2 plays a significant role in cardiolipin metabolism in Pru tachyzoites, thus maintaining mitochondrial homeostasis.

### The acyl-CoA-binding domain of ACBP2 is required for the intracellular growth of Pru parasites.

**FIG 6:**
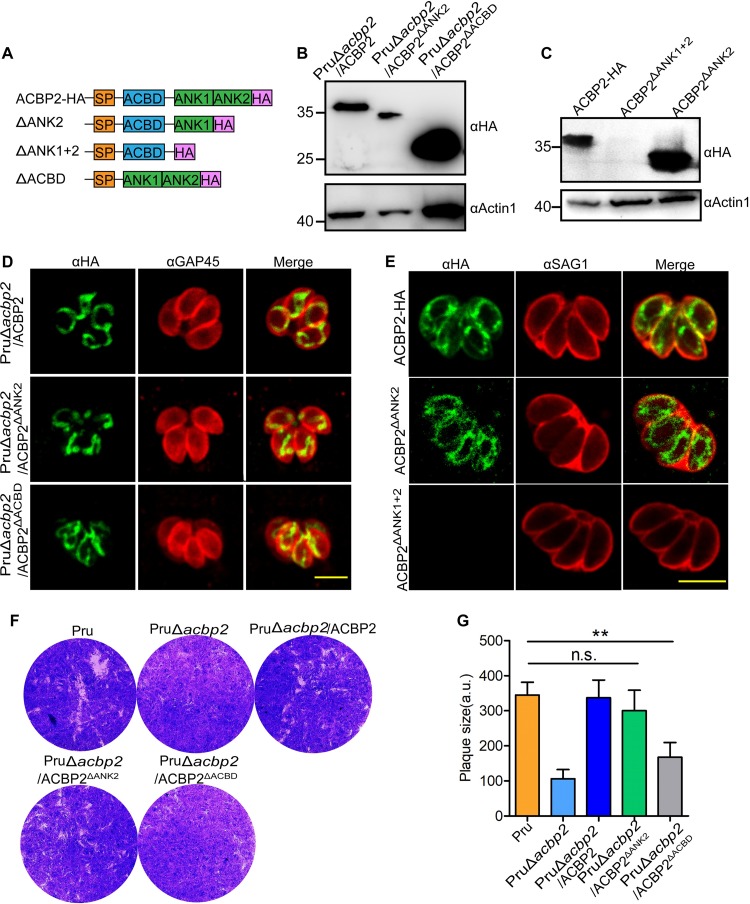
Complementation of strain PruΔ*acbp2* with ACBP2 mutants. (A) Schematic description of different ACBP2 mutants. (B) Western blotting confirmed the complementation of strain PruΔ*acbp2* with ACBP2^ΔANK2^ and ACBP2^ΔACBD^; anti-HA was used to detect the expression of ACBP2^ΔANK2^ and ACBP2^ΔACBD^; Actin1 was used as the loading control. (C) Western blotting confirmed the disruption of ANK2 and ANK1 + ANK2 domains of ACBP2 in strain RHΔ*ku80*, Actin1 was used as the loading control. (D) IFAs showed localization of ACBP2-HA, ACBP2^ΔANK2^, and ACBP2^ΔACBD^, demonstrating the presence of ANK2 or ACBD alone is not essential for the localization of ACBP2. Anti-HA was used to label ACBP2 mutants, while GAP45 was used as a marker of parasite membrane. Bar = 5 μm. (E) IFAs showed that disruption of the ANK2 domain resulted in the dispersion of ACBP2 in strain RHΔ*ku80*, while loss of ANK1 plus ANK2 completely blocked ACBP2 expression in strain RHΔ*ku80*. Anti-HA was used to label ACBP2 mutants, while SAG1 was used as a marker of parasite membrane. Bar = 5 μm. (F and G) Plaque assays of Pru, PruΔ*acbp2*, PruΔ*acbp2*/ACBP2, PruΔ*acbp2*/ACBP2^ΔACBD^, and PruΔ*acbp2*/ACBP2^ΔANK2^ strains showed that complementation of ACBP2^ΔANK2^ rather than ACBP2^ΔACBD^ restored the growth defect of strain PruΔ*acbp2*.

To confirm that ankyrin repeats and acyl-CoA-binding domain of ACBP2 play roles in the intracellular growth of Pru tachyzoites, PruΔ*acbp2* parasites were genetically complemented with an acyl-CoA binding domain (ACBD) truncation mutant, PruΔ*acbp2*/ACBP2^ΔACBD^, or an ankyrin repeat truncation mutant, PruΔ*acbp2*/ACBP2^ΔANK2^, and the results were confirmed by PCR (Fig. S4A and B) and Western blotting (Fig. 6A and B). IFA results showed that neither the ACBD nor the ANK2 truncation mutant affected the localization of ACBP2 in Pru tachyzoites (Fig. 6B). Complementation of ACBD mutant did not restore the PruΔ*acbp2* growth, while expression of ANK2 mutant rescued the growth defect of strain PruΔ*acbp2*, demonstrating the key role of the acyl-CoA binding domain of ACBP2 in the growth of Pru parasites (Fig. 6F and G).

10.1128/mBio.01597-18.4 FIG S4 PCR identification of disruption of ankyrin domains of ACBP2 in RHΔ*ku80* and complementation of strain PruΔ*acbp2* with ACBP2 mutants. (A) Illustration of complementation of strain PruΔ*acbp2* with ACBP2 mutants. Primers used to verify the genetic modification are indicated. (B) The replacements of the *uprt* locus of strain PruΔ*acbp2* by ACBP2^ΔANK2^ and ACBP2^ΔACBD^ were confirmed using primers S1 and S2. (C) Scheme illustrating the knock-in strategy to disrupt the ANK2 domain of ACBP2 by inserting HA tags at the C terminus. Primers used to verify the genetic modification are indicated. (D) The HA tagging at the locus of ankyrin repeats of ACBP2 by single homologous recombination was verified with PCR using primers K1 and K2. Download FIG S4, TIF file, 0.8 MB. Copyright © 2018 Fu et al. 2018 Fu et al. This content is distributed under the terms of the Creative Commons Attribution 4.0 International license.

However, we failed to complement PruΔ*acbp2* parasites with the ANK1 mutant. We hypothesized that ANK1 alone or in cooperation with ANK2 is involved in the expression or structural stability of ACBP2. To further reveal the roles of ankyrin repeats, we disrupted the ankyrin domains of ACBP2 in RHΔ*ku80* parasites by single homologous recombination and confirmed the disruption by PCR (Fig. S4C and D) and Western blotting (Fig. 6C). IFAs showed that loss of the ANK2 domain (ACBP2^ΔANK2^) resulted in dispersed localization on the mitochondrial membrane, while disruption of the two ankyrin repeats (ACBP2^ΔANK1 + 2^) blocked ACBP2 expression (Fig. 6E), demonstrating critical roles of ankyrin repeats in the expression of ACBP2. Taking the results collectively, these data revealed different localizations of ACBP2^ΔANK2^ in Pru and RHΔ*ku80* parasites and showed that the acyl-CoA-binding domain of ACBP2 is required for the intracellular growth of Pru tachyzoites.

### MAF1RHb1 rescued the growth defect of PruΔ*acbp2* parasites.

**FIG 7:**
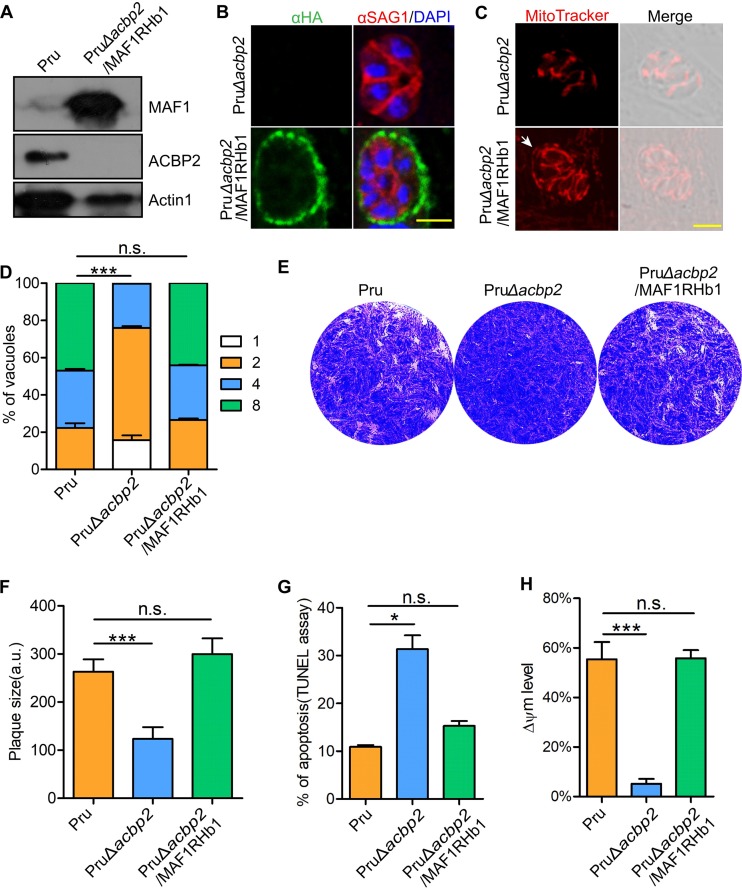
MAF1-mediated HMA rescued the growth defect of PruΔ*acbp2* tachyzoites. (A) Construction of PruΔ*acbp2* tachyzoites expressing an N-terminally HA-tagged MAF1RHb1 was confirmed by Western blot analysis using anti-HA and mouse anti-ACBP2 antibody. Actin1 was used as the loading control. (B) Expression of MAF1RHb1 in Pru parasites was further confirmed by IFA using antibodies against HA and SAG1. MAF1RHb1 is localized on the PVM. Bar, 5 μm. (C) MAF1RHb1-mediated HMA was assessed by IFA using MitoTracker. MAF1 could recruit host mitochondria around the *Toxoplasma* PVM in PruΔ*acbp2*/MAF1RHb1 parasites. Bar, 5 μm. (D) Proliferation assay showed that MAF1RHb1 expression in ACBP2-deficient parasites fully complemented the replication defect. The values are representative of the means ± SEM of results from three independent experiments. (E and F) Plaque assays were performed by infecting HFFs with 100 Pru, PruΔ*acbp2*, or PruΔ*acbp2*/MAF1RHb1 parasites and cultivating them for 12 days (E). Plaque area analysis demonstrated that MAF1RHb1 expression rescued the growth defects of the PruΔ*acbp2* parasites (F). The values are representative of the means ± SEM of results from two experiments. (G) Apoptosis of Pru, PruΔ*acbp2*, and PruΔ*acbp2*/MAF1RHb1 tachyzoites was assessed by the TUNEL assay. The results showed that MAF1RHb1 expression rescued the apoptosis defect caused by loss of ACBP2 in Pru parasites. Data are presented as the means ± SEM of results from three independent experiments. (H) ΔΨm was determined in Pru, PruΔ*acbp2*, and PruΔ*acbp2*/MAF1RHb1 tachyzoites by DiOC6(3) staining. FACS results showed that MAF1RHb1 expression rescued the decresed mitochondrial membrane potential caused by disruption of ACBP2 in Pru parasites. The error bars represent the standard errors of the means of results from three independent experiments.

Cardiolipins are major components of the mitochondrial inner membrane and could be synthesized and remodeled in mitochondria of both parasites and host cells. We wanted to determine whether host mitochondria provide cardiolipins directly or whether other lipids are involved in the cardiolipin metabolic pathways for parasites. However, HMA is present in type I and type III parasites but is missing in type II parasites. As MAF1 mediates *Toxoplasma* HMA (29), PruΔ*acbp2* tachyzoites expressing an N-terminally HA-tagged MAF1RHb1 were constructed (Fig. S5A) as previously reported (29). PCR, Western blotting, and immunofluorescence analysis revealed the expression of MAF1RHb1 in PruΔ*acbp2* tachyzoites (Fig. 7A and B; see also Fig. S5B). Confocal microscopy showed that expression of MAF1RHb1 in PruΔ*acbp2* tachyzoites was able to mediating HMA (Fig. 7C; see also Fig. S5C) and that ∼ 30% of PruΔ*acbp2*/MAF1RHb1 parasites recruited host mitochondria around the PVM (Fig. S5D), which was consistent with a previous report (29).

10.1128/mBio.01597-18.5 FIG S5 Expression of MAF1RHb2 in PruΔ*acbp2* tachyzoites induced host mitochondrial association (HMA). (A) Schematic representation of MAF1RHb1 expression in PruΔ*acbp2* parasites. (B) PCR identification of PruΔ*acbp2*/MAF1RHb1 tachyzoites. Primers M1 to M4 were designed for the verification of introduction of MAF1RHb1 into the *uprt* locus, while primers P1 and P2 (indicated in Fig. S2) were used for the verification of disruption of ACBP2 in strain PruΔ*acbp2*. (C) MAF1RHb1-mediated HMA was assessed by IFA using MitoTracker. MAF1RHb1 could recruit host mitochondria around the *Toxoplasma* PVM in PruΔ*acbp2*/MAF1RHb1 parasites. SAG1 was used as a marker for the periphery of parasites. Bar, 5 μm. (D) The percentage of parasitophorous vacuoles associated with host mitochondria was determined in PruΔ*acbp2* and PruΔacbp2/MAF1RHb1 parasites by counting at least 100 vacuoles after MitoTracker staining. Data are presented as the means ± SEM of results from three independent experiments. Download FIG S5, TIF file, 2.9 MB. Copyright © 2018 Fu et al. 2018 Fu et al. This content is distributed under the terms of the Creative Commons Attribution 4.0 International license.

To confirm our hypothesis that MAF1RHb1-mediated HMA could establish a metabolic link with T. gondii, we first investigated whether HMA in PruΔ*acbp2* tachyzoites restored the growth and intracellular proliferation of ACBP2-deficient tachyzoites. Strikingly, MAF1RHb1 expression in PruΔ*acbp2* tachyzoites successfully complemented the replication (Fig. 7D) and growth defects (Fig. 7E and F). Next, we found that expression of MAF1RHb1 restored the apoptosis rate (Fig. 7G) and mitochondrial potential (Fig. 7H) of strain PruΔ*acbp2*. These data suggest that MAF1RHb1 expression restored the mitochondrial health and intracellular growth of strain PruΔ*acbp2*.

### MAF1RHb1 complemented the cardiolipin metabolism defect of PruΔ*acbp2* parasites.

**FIG 8:**
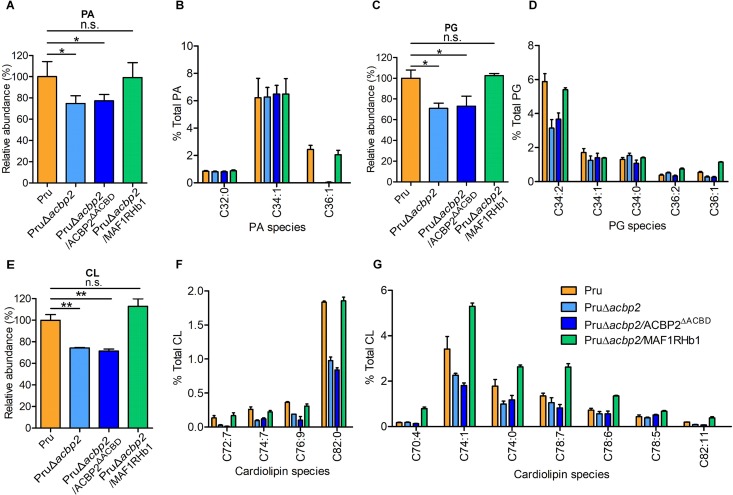
Lipidomic analysis revealed critical roles of ACBP2 and MAF1RHb1 in CL metabolism of parasites. Lipids extracted from Pru, PruΔ*acbp2*, PruΔ*acbp2*/ACBP2^ΔACBD^, and PruΔ*acbp2*/MAF1RHb1 tachyzoites were analyzed by HPLC-HRMS. (A, C, and E) Relative abundances for total PA, PG, and CL indicated that MAF1RHb1 rescued the defects of production of ∼30% PA (A), PG (C), and CL (E) in ACBP2-deficient parasites. (B, D, and F) HPLC-HRMS was used to identify in parasites 3 kinds of PA (C32:0, C34:1, and C36:1) (B) and 5 kinds of PG (C34:0, C34:1, C34:2, 36:1, and C36:2) (D), showing that disruption of ACBP2 resulted in reduced production of PA (C36:1) (B), PG (34:2) and PG (36:1) (D), and CL (72:7, 74:7, 76:9, and C82:0) (F), all of which could be complemented by expression of MAF1RHb1. (G) PruΔ*acbp2*/MAF1RHb1 tachyzoites produced more CL (74:0, 74:1, 74:4, 78:5, 78:6, 78:7, and 82:11) than the parental parasites. Error bars indicate standard deviations of results from three biological replicates.

We hypothesized that MAF1RHb1-mediated HMA might affect the lipid composition of strain PruΔ*acbp2*. To investigate the roles of MAF1RHb1 in lipid metabolism of strain PruΔ*acbp2*, we performed lipidomic analysis for Pru, PruΔ*acbp2*, PruΔ*acbp2*/ACBP2^ΔACBD^, and PruΔ*acbp2*/MAF1RHb1 parasites using HPLC-HRMS. The results show no obvious changes in the overall abundances of PC, PE, PI, and fatty acids among the different parasite strains (Fig. S6A to F). However, the overall abundances of both phosphatidic acid (PA) (Fig. 8A) and phosphatidylglycerol (PG) (Fig. 8C) were significantly reduced in PruΔ*acbp2* or PruΔ*acbp2*/ACBP2^ΔACBD^ parasites compared with Pru parasites, while MAF1RHb1 expression restored the abundance of PA and PG in strain PruΔ*acbp2*. Our analysis detected 3 PA molecular species (32:0, 34:1, and 36:1) (Fig. 8B) and 5 PG molecular species (34:2, 34:1, 34:0, 36:2, and 36:1) (Fig. 8D). We found that PA (36:1) abundance was obviously reduced in strain PruΔ*acbp2* and was restored followed by expression of MAF1RHb1 (Fig. 8B). Similarly, both PG (34:2) abundance and PG (36:1) abundance declined markedly in ACBP2-deficient parasites, while their abundances were increased in strain PruΔ*acbp2*/MAF1RHb1 and then returned to the normal level seen with the parental parasites (Fig. 8D). Next, we evaluated the cardiolipin abundances of different parasites and found that complementation of ACBP2^ΔACBD^ in strain PruΔ*acbp2* did not restore the cardiolipin overall abundance whereas expression of MAF1RHb1 rescued the cardiolipin abundance defect of strain PruΔ*acbp2* (Fig. 8E). Intensity analysis of 25 cardiolipin molecular species showed that MAF1RHb1 expression rescued the abundance of 4 cardiolipin molecular species (72:7, 74:7, 76:9, and 82:0) (Fig. 8F). In addition, MAF1RHb1 expression resulted in significantly higher abundances of 7 cardiolipin molecular species (70:4, 74:1, 74:0, 78:7, 78:6, 78:5, and C82:11) than were measured for the Pru parasites (Fig. 8G). There were 9 kinds of cardiolipins which were unaffected by either disruption of ACBP2 in Pru or complementation with MAF1RHb1 in strain PruΔ*acbp2* (Fig. S6G). However, MAF1RHb1 expression did not rescue the reduced abundances of 5 cardiolipin molecular species (74:9, 74:8, 78:11, 78:9, and 78:8) in strain PruΔ*acbp2* (Fig. S6H), demonstrating that MAF1RHb1 could not fully substitute for the role of ACBP2 in the production of these 5 cardiolipin molecular species. Taken together, these data revealed that MAF1RHb1 rescued most of the lipid metabolism defects caused by loss of functional ACBP2.

10.1128/mBio.01597-18.6 FIG S6 Neither loss of ACBP2 nor expression of MAF1RHb1 affected the overall abundances of PC, PE, PI, MLCL, and free fatty acids. (A to F) Relative abundances for total PC (A), PE (B), PI (C), PS (D), MLCL (E), and free fatty acids (F). These data demonstrated that neither disruption of ACBP2 nor expression of MAF1RHb1 changed the overall abundances of PC, PE, PI, MLCL, and free fatty acids. (G) Relative abundances of individual molecular species showed that neither disruption nor expression of MAF1RHb1 changed the relative abundances of CL (68:3, 68:5, 70:5, 74:2, 74:3, 74:4, 74:5, 80:1, and 82:3) in parasites. (H) MAF1RHb1 expression did not restore the reduced relative abundances of CL (74:8, 74:9, 78:8, 78:9, and 78:11) in PruΔ*acbp2* tachyzoites. Download FIG S6, TIF file, 1.7 MB. Copyright © 2018 Fu et al. 2018 Fu et al. This content is distributed under the terms of the Creative Commons Attribution 4.0 International license.

### Disruption of TgACBP2 caused attenuated virulence of Pru parasites in mice.

**FIG 9:**
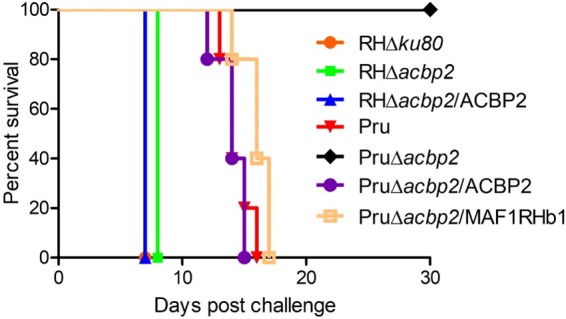
TgACBP2 is required for the virulence of type II parasites in mice. (A) Five BALB/c mice were infected with 2 × 10^4^ RHΔ*ku80*, RHΔ*acbp2*, RHΔ*acbp2*/ACBP2, Pru, PruΔ*acbp2*, PruΔ*acbp2*/ACBP2, or PruΔ*acbp2*/MAF1RHb1 tachyzoites, and their survival was assessed over 30 days. PruΔ*acbp2*-infected mice survived the initial challenge. Expression of both ACBP2 and MAF1RHb1 rescued the virulence defects of PruΔ*acbp2* parasites.

To investigate the roles of TgACBP2 in the virulence of tachyzoites, we inoculated the mice with 2 × 10^4^ RH, RHΔ*acbp2*, RHΔ*acbp2*/ACBP2, Pru, PruΔ*acbp2*, PruΔ*acbp2*/ACBP2, and PruΔ*acbp2*/MAF1RHb1 tachyzoites. All of the mice infected with RH, RHΔ*acbp2*, and RHΔ*acbp2*/ACBP2 tachyzoites died after 6 to 8 days (Fig. 9), demonstrating that ACBP2 knockout in type I parasites did not affect the virulence to mice. In contrast, mice infected with PruΔ*acbp2* tachyzoites remained alive 30 days postinfection (pi) (Fig. 9), while mice receiving 2 × 10^4^ Pru tachyzoites showed obvious symptoms of ruffled fur and ascites and died between day 13 and day 16 pi (Fig. 9). Importantly, complementation of ACBP2 restored the virulence of Pru tachyzoites, as mice infected with complemented parasites died between day 12 and day 15 pi (Fig. 9). Also, mice infected with PruΔ*acbp2*/MAF1RHb1 parasites died between day 14 and 18 pi (Fig. 9), demonstrating that MAF1 expression rescued the virulence defect of PruΔ*acbp2* parasites. Taking the results collectively, ACBP2 plays key roles in the virulence of Pru parasites in mice.

## DISCUSSION

Fatty acids, recognized as major building blocks of membranes, are initially activated with respect to the key intermediates LCACoAs. The intracellular trafficking and regulatory roles of LCACoAs rely mainly on ACBP (20). In this study, we identified an ankyrin repeat-containing ACBP2 which can bind LCACoAs and form acyl-CoA pool on mitochondria and showed that ACBP2 plays significant roles in stress response and cardiolipin metabolism, which is closely related to MAF1RHb1-mediated HMA.

Reportedly, ACBP could bind saturated and unsaturated C14 to C22 acyl-CoA esters with very high affinity (30–32), while we verified the binding activities of ACBP2 for C16:0-CoA. However, a wide range of acyl-CoAs (from middle chain to very long chain) are required to enable investigation of the substrate specificity of ACBP2 in future studies, which may provide a better understanding of functions of ACBP2. Moreover, binding activity analysis should be used to investigate ACBP1, SCP2, and other as-yet-uncharacterized acyl-CoA transporters in T. gondii, which may help constitute a complete trafficking network of acyl-CoA transporters for various acyl-CoA esters.

Adolase-1 of intracellular *Toxoplasma* translocates from the cytoplasm to pellicles in extracellular tachyzoites in response to changes in environmental [K^+^] and [Ca^2+^] (33). Here we showed that the translocation of ACBP2 from pellicle to the mitochondrial membrane is associated with [K^+^]- and [Ca^2+^]-induced dephosphorylation, which demonstrated that Ca^2+^ signaling may play roles in phosphorylation-dephosphorylation of ACBP2. Calcium-dependent protein kinases (CDPKs) are activated by Ca^2+^ signaling and thus phosphorylate various substrates, which play key roles in diverse cellular events, such as invasion, egression, motility, and protein development (22). Dynamic changes in [Ca^2+^] fluxes during these processes can lead to the activation of CDPKs, which may phosphorylate a multitude of downstream substrate proteins such as ACBP2. Further investigations are needed to monitor the phosphorylation of ACBP2 during the process of Ca^2+^ signaling-induced egress when the ion environment changes from high [K^+^] to high [Na^+^]. Although phosphoproteome research reveals over 10,000 phosphorylation sites in intracellular T. gondii (34), few data have been shown about the extracellular parasites. Obviously, ACBP2 represents the phosphorylated protein of extracellular parasites, while the specific phosphorylation sites should be further determined by mass spectrometry.

Phosphatidic acid serves as a mediator associated with microneme exocytosis, which demonstrates roles of lipids in signal transduction (35). Similarly, our work has revealed the mechanism of ACBP2 translocation and indicates that LCACoAs may be transported during the translocation as signaling molecules to regulate stress response. Actually, *Toxoplasma* has evolved to rapidly respond to extracellular stress outside host cells via the phosphorylation of eukaryotic initiation factor-2α (eIF2α) (36), the formation of RNA granules (37), and the induction of an apoptosis-like death pathway (24). Here, extracellular ACBP2-deficient parasites were shown to be vulnerable to high-[K^+^]-induced stress and displayed mitochondrial dysfunction and increased apoptosis rates, indicating that complexes of ACBP2-LCACoAs may act as messengers to transmit stress signals triggered by high extracellular [K^+^] to coordinate intracellular mitochondrial functions, while effects of phosphorylation-dephosphorylation of ACBP2 on the complex are to be further determined. However, one confusing problem is that RHΔ*acbp2* parasites exhibit no intracellular growth phenotype although RHΔ*acbp2* parasites naturally encounter high [K^+^] conditions in intracellular parasitophorous vacuoles. The natural process of invasion is very fast, and the parasite resides within the PV, which may protect the parasite from directly touching the host cell high [K^+^] environment. Therefore, the stress response to the ion environment changes from high [Na^+^] to high [K^+^] may be subtle during natural invasion and replication within the PV, while our study mimicked a long-lasting and directly operating stress environment by incubating extracellular parasites with high [K^+^] buffer.

Carnitine palmitoyl transferases function as transporters importing LCACoAs into mitochondria for β-oxidation and lipid biosynthesis. However, the *Toxoplasma* genome lacks genes encoding enzymes involved in β-oxidation or carnitine palmitoyl transferases. Interestingly, our demonstration of ACBP2 localization and active acyl-CoA-binding activities showed that ACBP2 may form an acyl-CoA ester pool on the mitochondrial membrane, thus providing the possibility of transporting LCACoAs to mitochondria for lipid metabolism but requiring further verification. Cardiolipin, containing two phosphate groups and four acyl chains, is specifically synthesized in mitochondria and constitutes the major mitochondrial inner membrane structure. Cardiolipin *de novo* synthesis starts from the conversion of mitochondrial PA to CDP-diacylglycerol (CDP-DAG), which provides a phosphatidyl group for PG synthesis. Finally, cardiolipin (CL) synthase catalyzes the transfer of the phosphatidyl group from CDP-DAG to PG. Recent studies have predicted a CL synthase candidate gene (TGGT1_309940), which is indicative of a potential *de novo* pathway for CL synthesis. Our lipidomic analysis of strain PruΔ*acbp2* showed that loss of ACBP2 resulted in significantly reduced abundance of PA, PG, and CL, which may associate the role of ACBP2 with CL *de novo* synthesis. Thus, we hypothesize that ACBP2 may provide acyl chains for PA and PG synthesized in mitochondria and plays roles in CL synthesis. Various cardiolipin species are formed by acyl remodeling following *de novo* cardiolipin synthesis (38). The remodeling relies mainly on the incorporation of acyl chains into monolysocardiolipin (MLCL), which is generated from deacylation of CL and reacylated for cardiolipin remodeling. We initially thought that loss of ACBP2 might change MLCL metabolism, while we found that ACBP2 disruption did not affect MLCL overall abundance or molecular species. One possibility is that MLCL mainly derives from those CLs that are independent of ACBP2, such as the unaffected 15 CL molecular species in strain PruΔ*acbp2*. In a word, ACBP2 plays important roles in lipid metabolism of parasite, especially cardiolipin metabolism, but needs further investigation of its roles in how to traffic acyl-CoAs to the lipid metabolic pathways localized in parasite mitochondria.

The different phenotypes of RHΔ*acbp*2 and PruΔ*acbp2* represented a confusing result, which could be elucidated by multiple pathways of cardiolipin acquirement. In previous studies, *Toxoplasma* was found to be capable of scavenging sphingolipids, phosphatidylcholine, and neutral lipids, such as triglycerides, from host cells across the PVM (28, 39, 40). Reportedly, PVMs of apicomplexan parasites, including *Toxoplasma*, *Plasmodium*, and *Eimeria*, are selectively permeable, allowing the passive, charge-independent diffusion of small molecules of up to 1.9 kDa into the PV (41–43). The greatest molecular weight of the cardiolipins we have identified is 1.6 kDa and is smaller than 1.9 kDa, which provides the possibility of the transport of cardiolipin across the PVM. Also, the intermediates and precursors involved in cardiolipin metabolism such as PA and PG might be able to pass the PVM. Hence, we hypothesize that type I parasites are capable of acquiring mature cardiolipins both by scavenging cardiolipins or the intermediates from host cells and by its own cardiolipin *de novo* synthesis and remodeling in mitochondria, while type II parasites cannot scavenge cardiolipins or the intermediates from host cells and depend on only its *de novo* synthesis pathway for cardiolipin synthesis. Therefore, disruption of ACBP2 in type II tachyzoites blocked the only source of mature cardiolipins and thus caused growth defects, while ACBP2-deficient type I parasites could alternatively utilize cardiolipins from host cells and maintain mitochondrial functions.

Considering that host mitochondria have emerged as significant sources of metabolites for *Toxoplasma* (39), we hypothesize that host mitochondria might affect lipid metabolism of parasite by providing various metabolites via host mitochondrial association (HMA), which is present in type I and III parasites but absent in type II parasites and is mediated by a family of genes encoding mitochondrial association factor 1 (MAF1) (29). Exogenous MAF1RHb1 expression in ME49 parasites can mediate HMA in type II parasites (44). As obvious recovery of growth and proliferation in strain PruΔ*acbp2*/MAF1RHb1 was observed, HMA may be closely related to the role of TgACBP2 in cardiolipin metabolism. Lipidomic analysis of strain PruΔ*acbp2*/MAF1RHb1 showed that MAF1RHb1 expression did not alter the overall abundances of PC, PE, PI, MLCL, and fatty acids but rescued most of the lipid metabolic defects such as reduced abundances of PA, PG, and CL caused by loss of functional ACBP2, showing strong evidence for the roles of host mitochondria in parasite lipid metabolism via HMA. Nearly 30% of the parasitophorous vacuoles of parasites expressing MAF1RHb1 recruit host mitochondria. We are very interested in the fates of the remaining 70% of parasitophorous vacuoles where parasites still express MAF1RHb1 but do not recruit host mitochondria. We hypothesize that *Toxoplasma* infection may cause host cell mitophagy, through which host cells segregate and remove unwanted or damaged mitochondria (45). When MAF1RHb1 mediates HMA, most of host mitochondria start mitophagy, releasing various lipids and other metabolites of mitochondria and presumably increasing local host lipid concentrations. In support of this hypothesis, host autophagy (including host lipophagy) is induced by *Toxoplasma* infection and is critical for the intracellular proliferation of the parasite (46). However, more evidence is needed to confirm the presence of mitophagy induced by MAF1RHb1-mediated HMA.

Taking the results collectively, TgACBP2 forms an acyl-CoA ester pool on mitochondria and is closely related to cardiolipin metabolism, while HMA rescued the growth and CL defects of PruΔ*acbp*2 parasites, establishing a metabolic association of host mitochondria with the parasites. Further functional studies on acyl-CoA-binding proteins will help build up networks of acyl-CoA partitioning and should shed more light on how the parasite acquires lipids from host cells by HMA, which may further our understanding of lipid metabolism in apicomplexans.

## MATERIALS AND METHODS

### Buffers.

The EC buffer consisted of the following: 120 mM NaCl, 1 mM CaCl_2_, 5 mM MgCl_2_, 25 mM HEPES-NaOH (pH 7.2), and 1 mg/ml bovine serum albumin (BSA).

The IC buffer consisted of the following: 142 mM KCl, 5 mM NaCl, 2 mM EGTA, 5 mM MgCl_2_, 25 mM HEPES-KOH (pH 7.2), and 1 mg/ml BSA.

### Host cells and *Toxoplasma* culture.

RHΔ*ku80* and Pru strains were used as parental parasites for the knockout of TgACBP2 in type I and type II parasites, respectively. Parasites were maintained by continuous passage in human foreskin fibroblasts (Cell Bank of the Chinese Academy of Sciences, Shanghai, China) using Dulbecco’s modified Eagle’s medium (DMEM) supplemented with 1% fetal bovine serum (FBS), 10 units/ml penicillin, and 100 mg/ml streptomycin at 37°C and 10% CO_2._ Intracellular parasites were harvested into IC buffer after 48 h of culture in HFF for 2 h of incubation, while extracellular parasites were harvested into EC buffer after natural egress for 2 h of incubation. Parasite transfection was performed as previously described (47).

### Construction of transgenic parasite lines.

To construct TgACBP2-HA parasites, the 3′ regions of TgACBP2 were amplified from genomic DNA (gRNA) of RHΔ*ku80* parasites with primers 1 and 2 (listed in Table S1 in the supplemental material), followed by ligation with pLIC-3×HA-dihydrofolate reductase (DHFR) plasmid digested with EcoRV-HF and ApaI by seamless cloning kit (Vazyme). The resulting vectors were linearized with PacI, transfected into RHΔ*ku80* parasites, and selected on pyrimethamine. The monoclones were identified by PCR followed by sequencing.

10.1128/mBio.01597-18.7 TABLE S1 List of primers used in this study. Primers 1 to 23 were used for the construction of genetically modified parasites as indicated in Materials and Methods. Primers P1 to P7 were designed for the verification of disruption of ACBP2 in RHΔ*ku80* parasites. Primers M1 to M4 were used for the verification of introduction of MAF1RHb1 into *uprt* locus. Primers K1 and K2 were used for the identification of ankyrin repeat-deficient parasites of RHΔ*ku80*. Primers S1 and S2 were designed for the identification of complementation of strain PruΔ*acbp2* with ACBP2 mutants. Download Table S1, DOCX file, 0.02 MB. Copyright © 2018 Fu et al. 2018 Fu et al. This content is distributed under the terms of the Creative Commons Attribution 4.0 International license.

10.1128/mBio.01597-18.8 TABLE S2 Cardiolipin species identification in *Toxoplasma* by LC-HRMS. Cardiolipin species (25 kinds) were identified in the HPLC-HRMS results. The first column show the total number of carbons and unsaturation of each kind of cardiolipin, whose corresponding *m*/*z* data are shown in the second column, while the formula of each kind of cardiolipin is described in the third column. Download Table S2, DOCX file, 0.02 MB. Copyright © 2018 Fu et al. 2018 Fu et al. This content is distributed under the terms of the Creative Commons Attribution 4.0 International license.

To construct plasmid pCRISPR-CAS9-ACBP2, upstream and downstream fragments containing gRNA sequences were amplified with primers 3 and 4 and primers 5 and 6, respectively, followed by ligation into the AvrII and KpnI sites of pSAG1-CAS9-U6gRNA (UPRT) by seamless cloning.

To construct ACBP2-deficient parasites, the 3′ flank and 5′ flank of TgACBP2 were amplified using primers 7 and 8 and primers 9 and 10, respectively, from genomic DNA of strain RHΔ*ku80* parasites. CAT-RFP (CAT-red fluorescent protein) and the vector backbone were amplified with primers 11 and 12 and primers 13 and 14 from pTCR-CD. Finally, plasmid p5′ACBP2-TCR-3′ACBP2-CD was completed by seamless cloning using the four fragments. After linearization using NotI, the plasmid was transfected into RHΔ*ku80* parasites and with pCRISPR-CAS9-ACBP2 into Pru parasites. Finally, the transfected parasites were selected on chloromycetin and 5-flucytosine for positive and negative screening, respectively. For the disruption of ankyrin repeats of ACBP2 in strain RHΔ*ku80*, we adopted a knock-in strategy based on single homologous recombination and thereby constructed pLIC-ACBP2^ΔANK2^-3×HA-DHFR and pLIC-ACBP2^ΔANK1 + 2^-3×HA -DHFR plasmids to replace the ANK2 and ANK1 + 2 locus with 3×HA tags. The 5′ flanks chosen for the disruption were amplified using primers 15 and 16 and primers 15 and 17, followed by ligation with pLIC-3×HA-DHFR plasmid digested with EcoRV-HF and ApaI by the use of a seamless cloning kit (Vazyme). The resulting vectors were linearized with PacI, transfected into RHΔ*ku80* parasites, and selected on pyrimethamine. The monoclones were identified by PCR followed by sequencing.

To complement ACBP2-deficient parasites, we first constructed *uprt* locus-targeted homologously recombinant plasmid p5′UPRT-Tubulin promoter-HA-DHFR-3′UPRT. The TgACBP2 full ORF was amplified with primers 18 and 19 from cDNA of RHΔ*ku80* strain parasites, while ORFs of ACBP2^ΔACBD^, ACBP2^ΔANK1^, and ACBP2^ΔANK2^ were amplified using primers 18 and 19 from the plasmids, which contain synthesized sequences of ACBP2^ΔACBD^, ACBP2^ΔANK1^, and ACBP2^ΔANK2^. All these ACBP2 ORFs were ligated into ApaI and NheI sites of p5′UPRT-Tubulin promoter-HA-DHFR-3′UPRT to produce p5′UPRT-ACBP2-HA-DHFR-3′UPRT, p5′UPRT-ACBP2^ΔACBD^-DHFR-3′UPRT, p5′UPRT-ACBP2^ΔANK1^-DHFR-3′UPRT, and p5′UPRT-ACBP2^ΔANK2^-DHFR-3′UPRT. Finally, the fragments chosen for transfection with clustered regularly interspaced short palindromic repeat (CRISPR)-Cas9 plasmids were amplified using primers 20 and 21 and transfected with pSAG1-CAS9-U6gRNA (UPRT) into strain RHΔ*acbp2* or strain PruΔ*acbp2* (1:5 mass ratio). All the transfected parasites were selected on pyrimethamine and floxuridine (FUDR) for positive and negative screening, respectively. The monoclones were identified by PCR followed by sequencing.

To express MAF1 in strain PruΔ*acbp*2 parasites, the *MAF1RHb1* gene was synthesized as previously described (29), amplified with primers 22 and 23, and ligated into ApaI and NheI sites of p5′UPRT-Tubulin promoter-HA-DHFR-3′UPRT to produce plasmid p5′UPRT-MAF1-HA-DHFR-3′UPRT. After linearization by the use of BlpI, the plasmid was transfected with pSAG1-CAS9-U6gRNA (UPRT) into PruΔ*acbp2* parasites. The monoclones were identified by PCR followed by sequencing.

### Fluorescence-based binding kinetics.

This fluorometric assay was carried out as previously described (48). The fluorescence-based assay was based on a structural feature of nitrobenzoxadiazole (NBD), which is nearly nonfluorescent in aqueous solution but can produce increased fluorescence in a polar environment such as the binding pocket of ACBP. In this assay, the emission of NBD-labeled palmitoyl-CoA (NBD-C16:0-CoA) upon binding to TgACBP2 was measured using multifunctional microplate reader SpectraMax M5 with a 540-nm wavelength for emission and 460-nm wavelength for excitation. A binding kinetics assay was performed in 96-well white plates using a mixture consisting of 0.1 μM TgACBP2-GST and 0 nM, 0.015625 μM, 0.03125 μM, 0.0625 μM, 0.125 μM, and 0.25 μM NBD-C16:0-CoA, with PBS added to achieve a final volume of 100 μl. The reaction was kept at room temperature for 5 min, and fluorescence levels were determined. Data were plotted. and the binding constant K was calculated in GraphPad Prism 5.

### Yeast complementation assay.

Yeast complementation assays were performed as previously described (49). Briefly, the TgACBP2 gene was inserted into yeast expression vector p405ADH1. Yeast strain BY4741 lacking ACBP (*acb1*Δ) was transformed with p405ADH1-TgACBP2 or empty vector. Vacuole structures of the parental, *acb1*Δ, *acb1*Δ+vector, and *acb1*Δ+TgACBP2 yeasts were observed by fluorescence microscopy using fluorescent dye FM1-43. Yeasts with single-lobed or multilobed vacuoles were counted using a Leica confocal microscope system (TCS SP52; Leica, Germany).

### Immunoblotting and immunofluoresence assays.

For antibodies (Abs), mouse anti-HA monoclonal Ab (MAb) was purchased from Sigma. Mouse anti-F1bATPase (MAb) was kindly provided by Peter J. Bradley, while mouse anti-CytC, anti-profilin, anti-ACBP2, anti-Actin1, anti-ACP, and anti-IMC1 and rabbit anti-SAG1 and anti-GAP45 were all polyclonal antibodies prepared by our laboratory.

For Western blotting assays, parasites were disrupted by sonication on ice followed by SDS-PAGE. The primary antibodies used in this study were mouse anti-HA (MAb, 1:100), anti-CytC (1:200), anti-profilin (1:200), anti-ACBP2 (1:500), and anti-Actin1 (1:2,000). For the preparation of mouse anti-ACBP2 antibody, full-length His-tagged ACBP2 was expressed in E. coli and used to immunize mice after purification.

For Phos-tag gel electrophoresis, which involves the use of a Phos-tag biomolecule that specifically binds phosphorylated proteins and retards their migration in the gel, Phos-tag gel electrophoresis was carried out according to the manufacturer’s instructions (Wako Chemicals, USA). Briefly, 50 μM Phos-tag and 100 μM MnCl_2_ were added to 12% (wt/vol) acrylamide resolving gel and the gel was run at a constant voltage at room temperature. The gel was soaked in a general transfer buffer containing 1 mM EDTA for 10 min with gentle agitation, followed by one wash in transfer buffer without EDTA performed for 20 min before being transferred to a polyvinylidene difluoride (PVDF) membrane for immunoblotting using anti-ACBP2 antibody.

For immunofluorescence assays, parasite-infected HFFs or freshly released parasites were fixed by the use of 4% paraformaldehyde (PFA) before being permeabilized with 0.25% Triton X-100–PBS. The samples were then blocked by the use of 3% BSA–PBS and incubated with primary mouse anti-HA (1:50), anti-F_1_bATPase (1:100) (kindly provided by Peter J. Bradley), anti-ACP (1:50) or rabbit anti-SAG1 (1:100), and anti-GAP45 (1:100) for 1 h. All samples were stained with Hoechst and imaged with a Leica confocal microscope system (TCS SP52, Leica, Germany). MitoTracker staining was performed as previously described (44). Parasite-infected HFFs were cultured in media containing 50 nM MitoTracker Red CMXRos for 30 min at 37°C. After two washes, samples were cultivated for a further 4 h. Finally, samples were processed as described for the IFA procedure.

### Cell fractionation.

Cell fractionation was performed as previously described (50). Freshly released parasites were resuspended in 25 mM morpholinepropanesulfonic acid (MOPS)-KOH buffer (pH 7.0) (containing 5 mM MgCl_2_, protease inhibitors, and 25 mM, 150 mM, or 300 mM KCl), PBS, PBS–1 M NaCl, or PBS–0.1 M Na_2_CO_3_ (pH 11.5) and were disrupted by sonication on ice. Samples were centrifuged for 20 min at 14,000 rpm at 4°C. The pellets were dissolved in the same buffers containing 1% Triton X-100. Total lysates, pellets, or supernatants were detected by Western blotting.

### Alkaline phosphatase analysis.

ACBP2-HA intracellular or extracellular parasites were collected followed by sonication. Pellets and supernatants were separated by centrifugation. ACBP2-HA was immunoprecipitated from the supernatants and incubated with conjugated anti-HA antibody agarose beads, followed by three washes. The beads were then incubated with reaction buffer in the presence or absence of 1 U alkaline phosphatase for 30 min or 1 h at 37°C before termination of the reaction by boiling.

### Phenotypic assays. (i) Plaque assays.

HFFs growing in 6-well plates were infected with parasites for 8 or 12 days followed by fixation with PFA and crystal violet staining.

### (ii) Proliferation assays.

A total of 10^6^ freshly isolated parasites were inoculated onto 12-well plates for 24 h following IFA using rabbit anti-SAG1. Numbers of parasites per vacuole were determined by counting at least 100 vacuoles.

### (iii) Evaluation of mitochondrial functions.

To detect reactive oxygen species (ROS) levels, DCFH-DA was added to cells. ROS could react with nonfluorescent DCFH-DA, which is rapidly oxidized to the highly fluorescent 2’,7’-dichlorodihydrofluorescein (DCF). The proportion of fluorescent cells was measured by flow cytometry. In this assay, freshly harvested parasites were incubated with 30 μM DCFH-DA and 100 nM MitoTracker at 37°C for 1 h and then resuspended in 100 μl PBS after two washes.

Mitochondrial membrane potential was measured using DiOC6(3) as previously described (51). Briefly, freshly isolated parasites were incubated with 5 nM DiOC6(3) and 100 nM MitoTracker at 37°C for 1 h followed by two washes in PBS. Finally, 100-μl suspensions of parasites were analyzed. All data were collected by the use of an LSRII cytometer (BD Biosciences) and analyzed by FlowJo 7.6.

### (iv) Extraction of cytosolic proteins and detection of cytochrome *c* release.

A total of 1 × 10^8^ tachyzoites were harvested in 250 μl chilled extraction buffer (250 mM sucrose, 1 mM EDTA, 50 mM Tris-HCl, 1 mM dithiothreitol [DTT], 1 mM phenylmethylsulfonyl fluoride [PMSF], 1 μM protease cocktail, pH 7.4) followed by repeated freeze-thaw cycles in liquid nitrogen and were lysed by soft sonication, followed by centrifugation at 100,000 × *g* for 1 h at 4°C. The supernatant was collected and used as a cytosolic extract. Before detection, anti-F_1_bATPase was used to monitor the level of mitochondrial contamination in the supernatant.

### (v) TUNEL assay.

TUNEL assays were performed using an apoptosis detection kit (Vazyme Biotech) as previously reported (25). Briefly, freshly egressed tachyzoites were treated with IC buffer as a prostimulus for 2 h, followed by fixation on coverslips. After permeabilization, parasites were incubated in TUNEL reaction mix with TdT enzyme and nuclei were stained with Hoechst (Sigma, USA). SAG1 was used as a marker of parasite shape. A total of 100 parasites were counted to determine the number of TUNEL-positive parasites.

### (vi) Lipidomic analysis using HPLC-HRMS.

Lipids were extracted from parasites as previously reported (52). A total of 2 × 10^8^ parasites were resuspended in chloroform-methanol-water (2.4 ml; 1:1:0.4 [vol/vol]) followed by sonication, while C56:0 served as an internal standard and was added to the parasites prior to the extraction. After centrifugation at 12,000 × *g* for 15 min, the lower phase was collected, dried, and resupended in 100 μl methanol. First, 2-μl samples were analyzed by the use of an LC-20ADXR liquid chromatograph (Shimadzu). The HPLC mobile phases included solution A (acetonitrile-water-methanol, 20:60:20 [vol/vol/vol]), 5 mM ammonium acetate) and solution B (isopropanol-acetonitrile, 90:10 [vol/vol], 5 mM ammonium acetate). The gradient was from 60% solution A to 100% solution B in 25 min and maintained at 100% solution B for 45 min in a Kinetex C_18_ column (2.1 mm by 100 mm, 2.6-μm pore size) at a flow rate of 0.4 ml/min at 40°C. MS analysis was performed on an AB 5600^+^ Quad Time Of Flight (QTOF) system (Sciex). The electrospray ionization (ESI) source settings were as follows: curtain gas, 35; gas temperature, 500°C; collision energy, −40; declustering potential, −100; accumulation time, 0.25 s; TOF MS, *m*/*z* 100 to 2,000; polarity for fatty acids, MLCL, CL, PI, PS, PA, and PG, negative; polarity for PC and PE, positive. Data were normalized to the abundance of C56:0 and analyzed by the use of Analyst TF 1.6 (Sciex).

### T. gondii mouse infection.

BALB/c mice were infected with 2 × 10^4^ RH, RHΔ*acbp2*, RHΔ*acbp2*/ACBP2, Pru, PruΔ*acbp2*, PruΔ*acbp2*/ACBP2, or PruΔa*cbp2*/MAF1RHb1 tachyzoites. The period for observing the survival was more than 5 weeks.

### Ethics statement.

The experiments performed were in strict accordance with the recommendations of the Guide for the Care and Use of Laboratory Animals of the Ministry of Science and Technology of China. All experimental procedures were approved by the Institutional Animal Care and Use Committee of China Agricultural University (under the certificate of Beijing Laboratory Animal employee ID CAU20161210-2).

### Statistical analysis.

Graphs and statistical analyses were made using GraphPad Prism (GraphPad, San Diego, CA). All data were analyzed with the two-tailed Student's *t* test. *P* values are represented in the figures as follows: *, *P* = <0.05; **, *P* = <0.01; ***, *P* = <0.001; n.s., not significant. We consider all *P* values of <0.05 to be significant.
