# Supplementary figures and images for: Comprehensive Characterization of Toxoplasma Acyl Coenzyme A-Binding Protein TgACBP2 and Its Critical Role in Parasite Cardiolipin Metabolism
Source: mBio. 2018 Oct 23;9(5):e01597-18. doi: 10.1128/mBio.01597-18 (PMC6199492; doi:10.1128/mBio.01597-18)

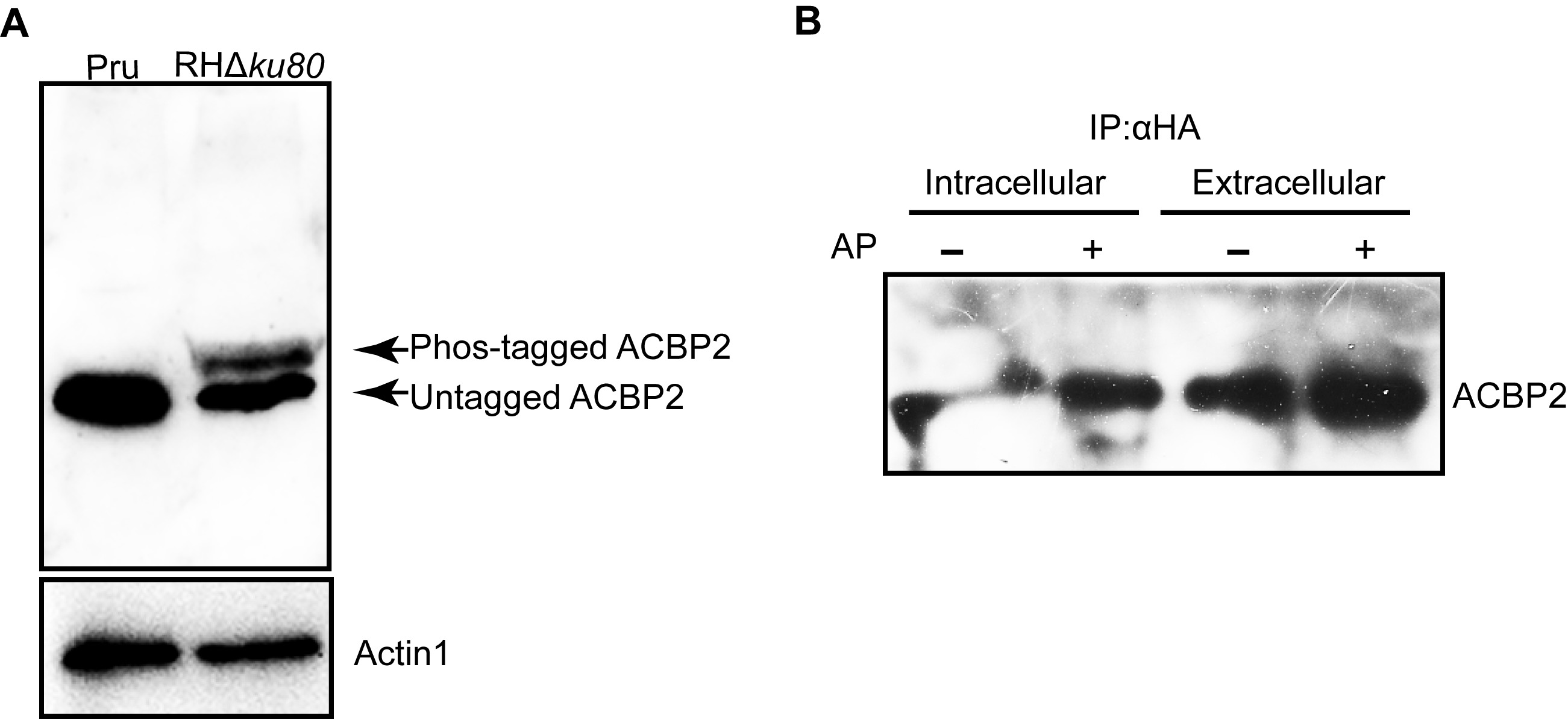

Supplement: FIG S1 [file mbo005184118sf1.tif]

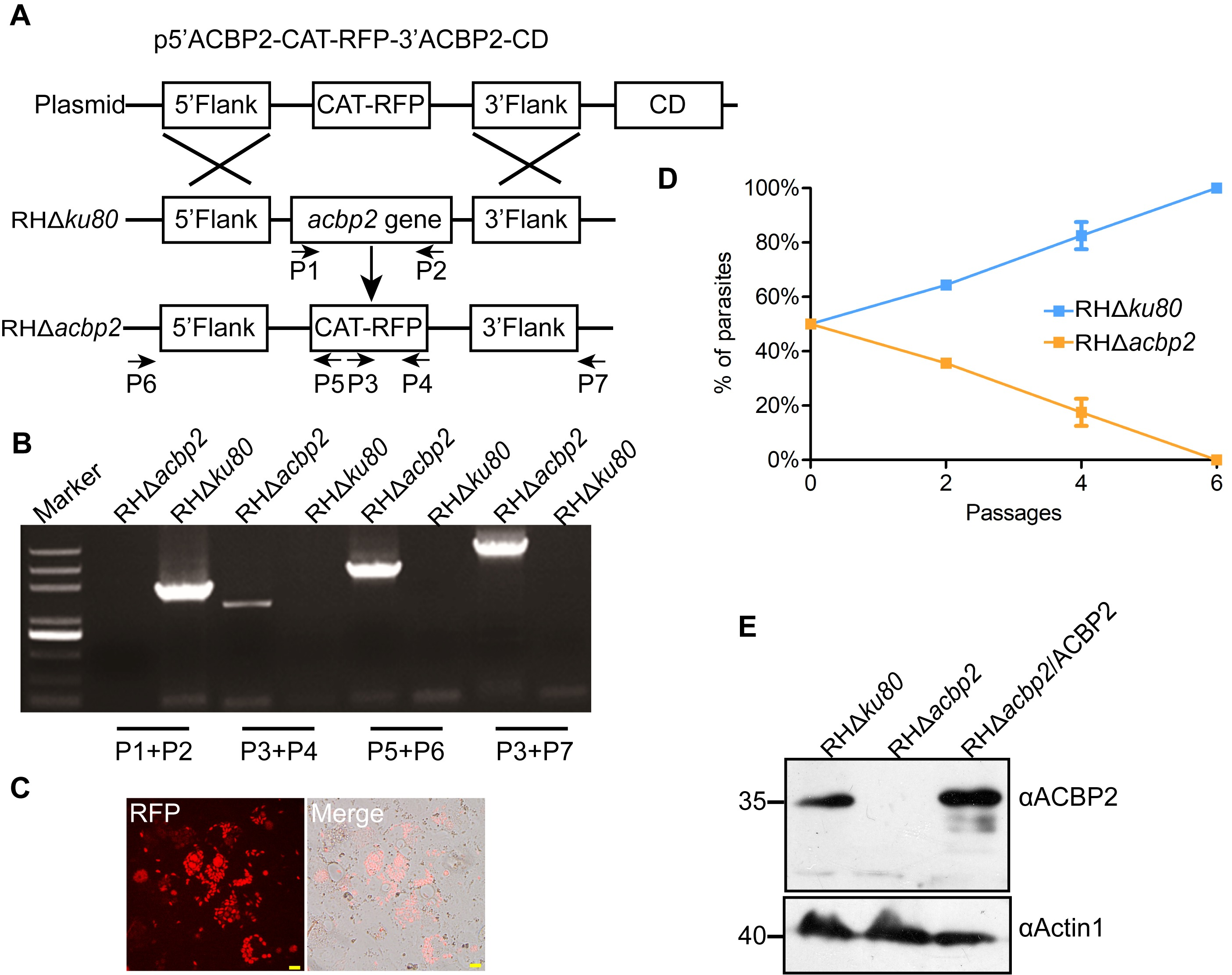

Supplement: FIG S2 [file mbo005184118sf2.tif]

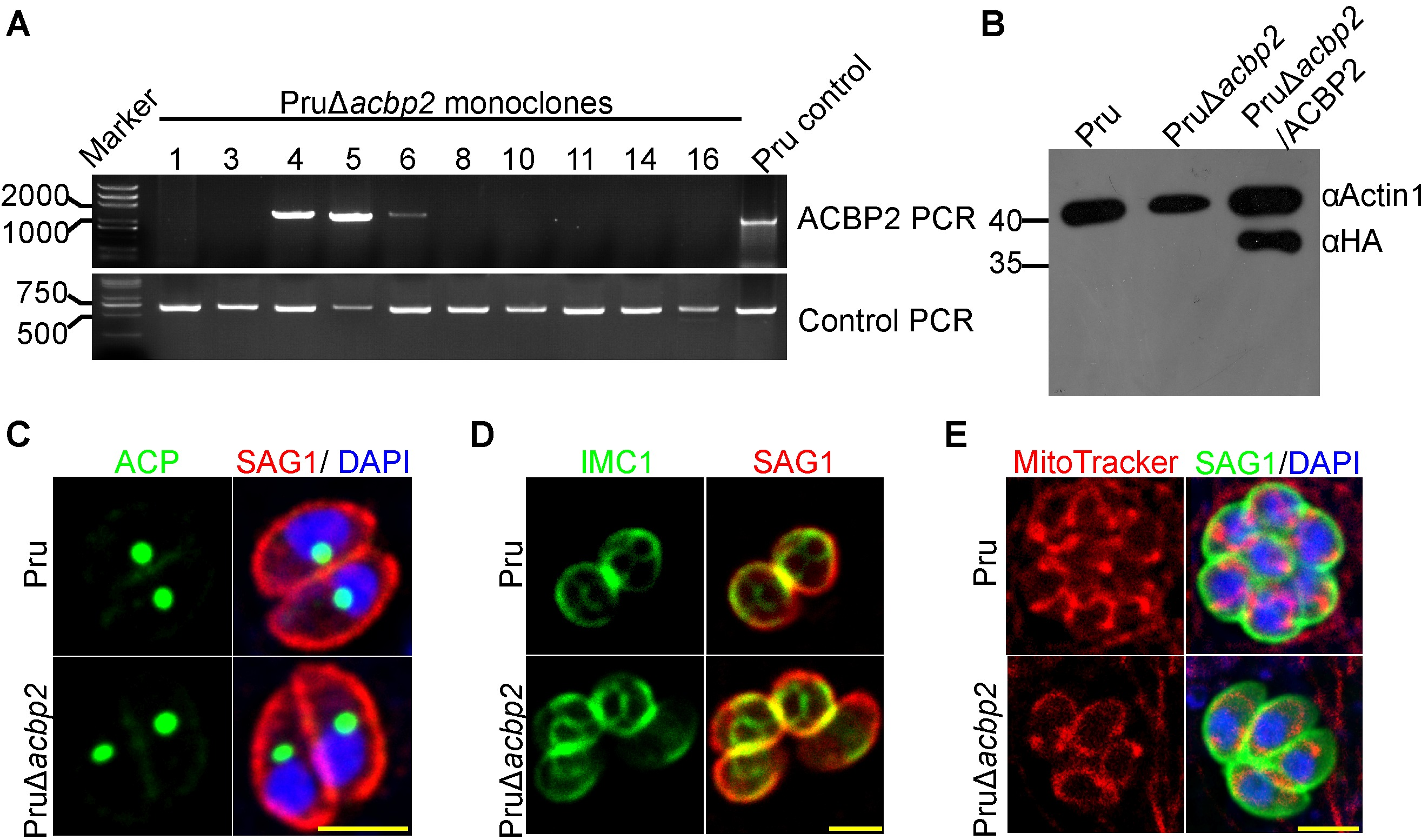

Supplement: FIG S3 [file mbo005184118sf3.tif]

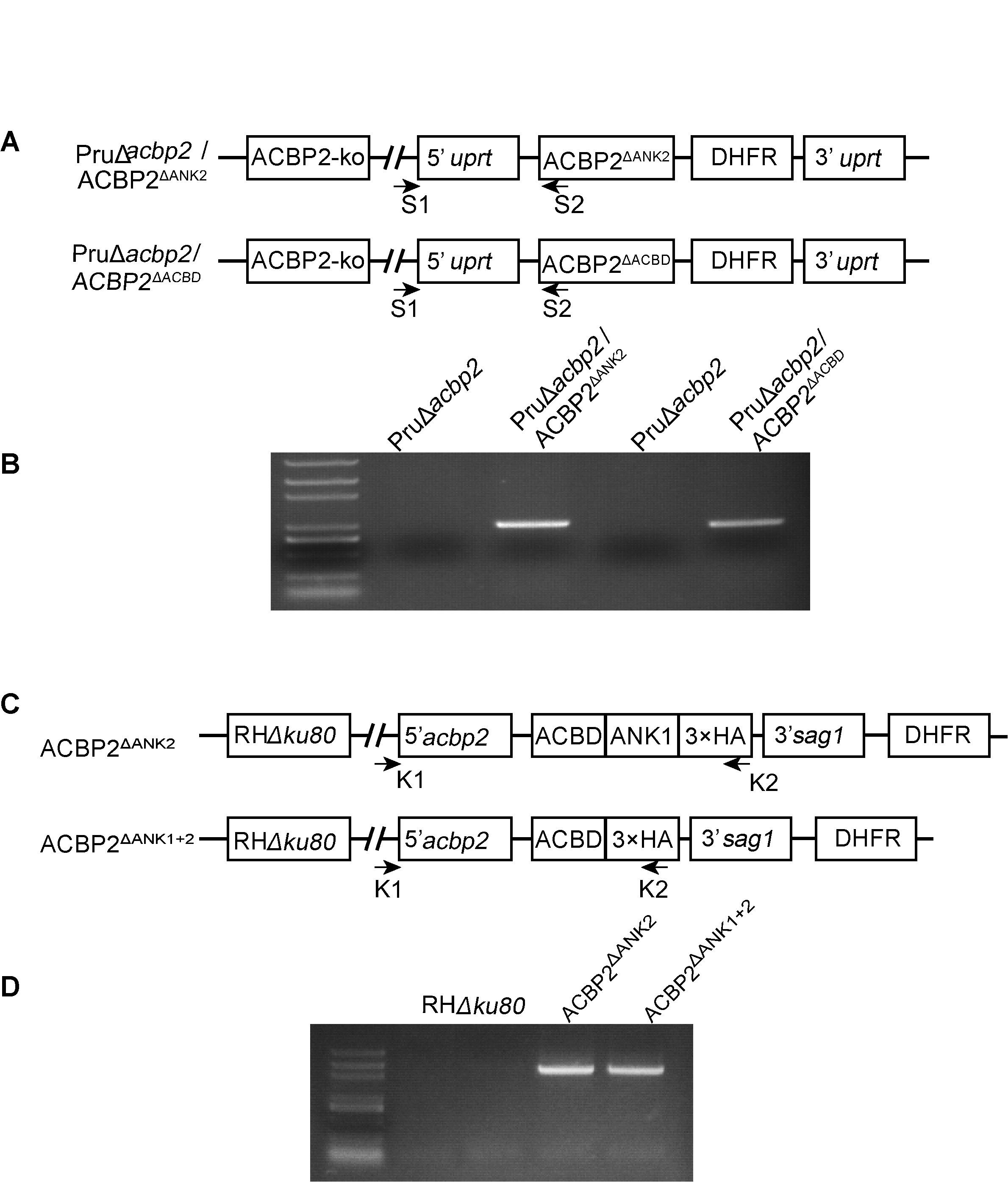

Supplement: FIG S4 [file mbo005184118sf4.tif]

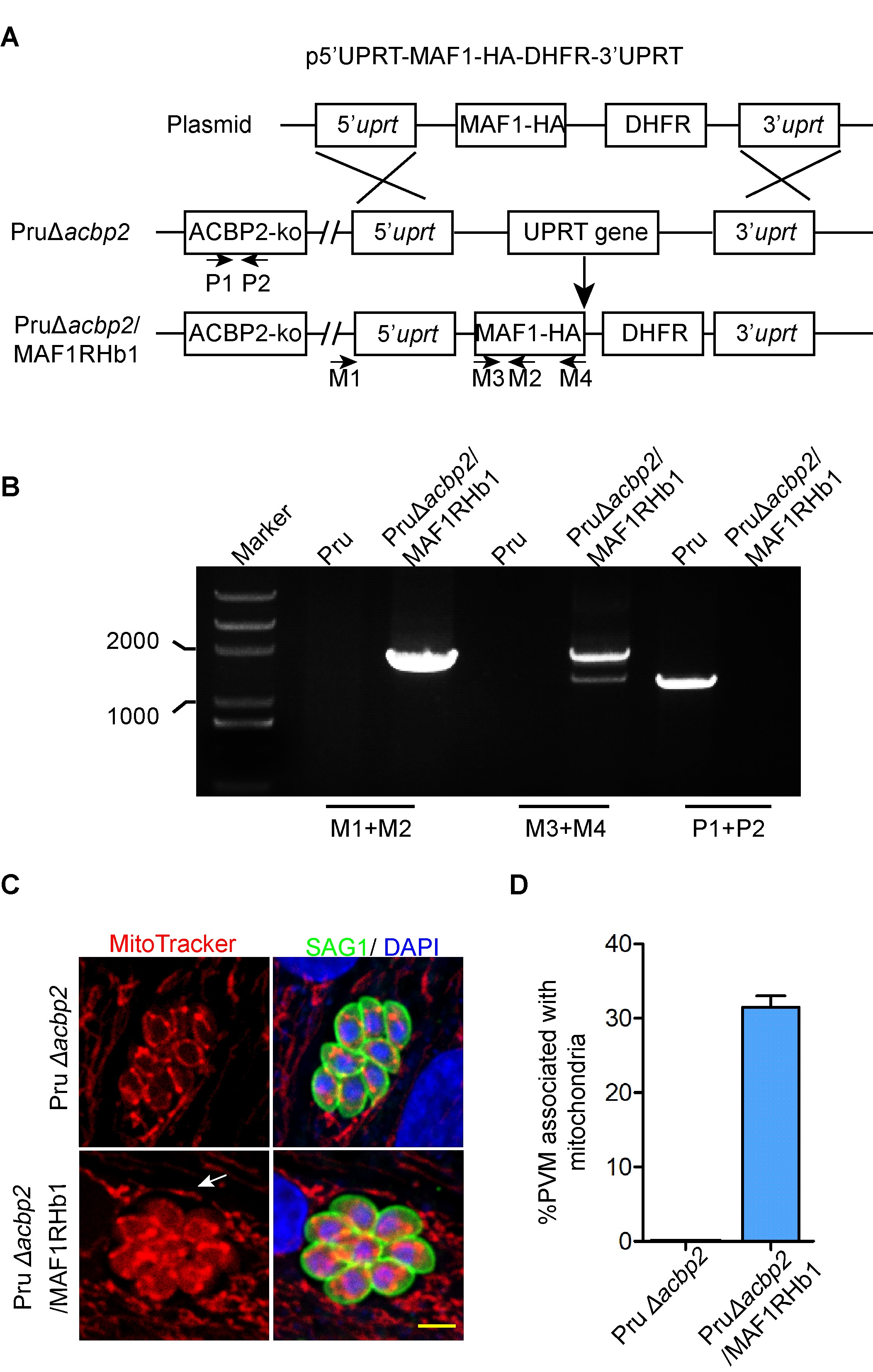

Supplement: FIG S5 [file mbo005184118sf5.tif]

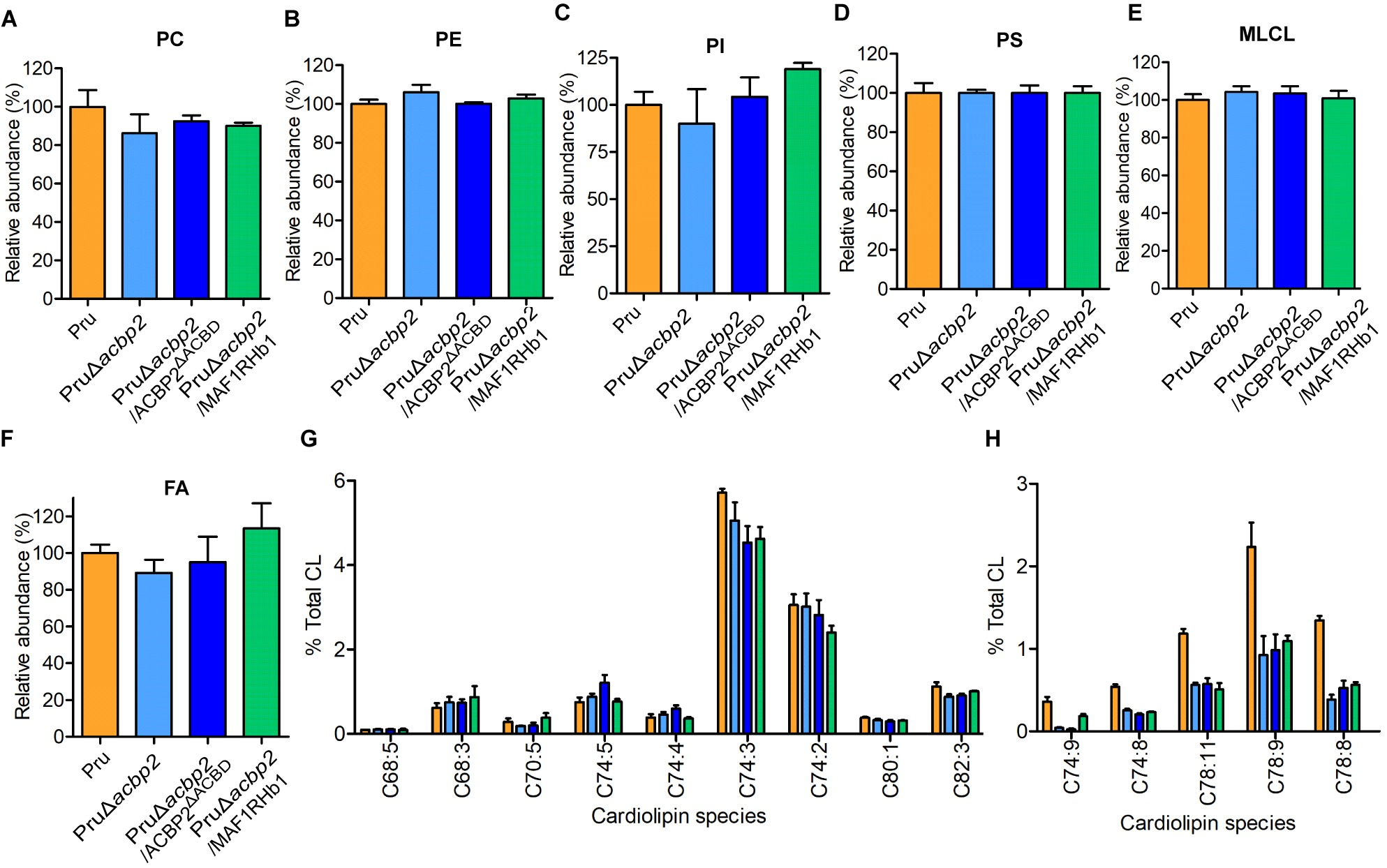

Supplement: FIG S6 [file mbo005184118sf6.tif]
