# Supplementary material for: Comprehensive Characterization of Toxoplasma Acyl Coenzyme A-Binding Protein TgACBP2 and Its Critical Role in Parasite Cardiolipin Metabolism
Source: mBio. 2018 Oct 23;9(5):e01597-18. doi: 10.1128/mBio.01597-18 (PMC6199492; doi:10.1128/mBio.01597-18)
Supplement: TABLE S1 [file mbo005184118st1.docx]

**Table S1** List of primers used in this study.

| No. | Primer sequence (5’-3’) |
| --- | --- |
| 1 | TCCAATTTAATTAAGATATC TCTATGCTGCTCAGGAACGC |
| 2 | CCTCCACTTCCAATGGGCCC AGTAGCTTTTGAGGCGGTGA |
| 3 | GCCAATATTGCATCCTAGG |
| 4 | CTTACCTTTTTCAAGGAGAA AACTTGACATCCCCATTTAC |
| 5 | TCGAGGGGGGGCCCGGTACC |
| 6 | TTCTCCTTGAAAAAGGTAAG GTTTTAGAGCTAGAAATAGC |
| 7 | GATATCTCTAGTGGATCCC |
| 8 | GCGAATTGGGTACCGGGCCC TGAAGGACTTTGCTTGGTC |
| 9 | TGGTTGAAAGAAGCAGGGT |
| 10 | GGGATCCACTAGAGATATC AAGTCGTATAAACGAACTAT |
| 11 | CGCGGGACATGCATACTAGT CCTCTTCTTTTACGGCTGCG |
| 12 | CGCGGGACATGCATACTAGT GGTTTGAGGTTCGTGGAGC |
| 13 | ACCCTGCTTCTTTCAACCAAA GCTTGATATGCATGTCCGC |
| 14 | CGCGGGACATGCATACTAGT GCGAAGACTCCGTTTCTGA |
| 15 | TCCAATTTAATTAAGATATC CACCCTGCTTCTTTCAACCA |
| 16 | CCTCCACTTCCAATGGGCCC GGCGTTTACGTCAGCCC |
| 17 | CCTCCACTTCCAATGGGCCC ATCATTTCTTGCAGTCACCAGC |
| 18 | TAGATCGATCTAAAGGGCCC ATGGCGAGGCCTGTACATCT |
| 19 | ACATCGTAAGGATAGCTAGC AGTAGCTTTTGAGGCGGTG |
| 20 | CGAGGGCCATATCGATCCCA |
| 21 | ACGCCAACTTCCCTACACG |
| 22 | TAGATCGATCTAAAGGGCCC ATGTGGCGCATCTGGAGATG |
| 23 | ACATCGTAAGGATAGCTAGC TCAGTCCAGCATGCTAGCCA |
| P1 | ATGGCGAGGCCTGTACATCTTG |
| P2 | AGTAGCTTTTGAGGCGGTG |
| P3 | AGAGTCCTCCTTGTTCCATT |
| P4 | TGATTAACTACTGGGAGCCG |
| P5 | CTTCGCTGCCCTTTCTTGT |
| P6 | CAACTGACTGAAATGCCTC |
| P7 | AAACCCGAGAGAACTCGAG |
| M1 | TCTTGTCCCTGGGTGTTTC |
| M2 | TTTGGTGGATGTCTTCTGC |
| M3 | ATGTGGCGCATCTGGAGATG |
| M4 | CGGCGATCAACATCAGCCAG |
| K1 | CTGTGTTTATCTTGTGAGGC |
| K2 | ACGTCGTACGGGTACCTAGG |
| S1 | TGCAGTGTGGCGGTAAGACGAG |
| S2 | AGATGTACAGGCCTCGCCAT |

Primers 1-23 were used for the construction of genetically modified parasites as indicated in Materials and Methods. Primers P1-P7 were designed for the verification of disruption of ACBP2 in RHΔ*ku80* parasites. Primers M1-M4 were used for the verification of introduction of MAF1RHb1 into *uprt* locus. Primers K1-K2 were used for the identification of ankyrin repeats-deficient parasites of RHΔ*ku80*. Primers S1-S2 were designed for the identification of complementation of PruΔ*acbp2* with ACBP2 mutants.
