# Supplementary material for: Comprehensive Characterization of Toxoplasma Acyl Coenzyme A-Binding Protein TgACBP2 and Its Critical Role in Parasite Cardiolipin Metabolism
Source: mBio. 2018 Oct 23;9(5):e01597-18. doi: 10.1128/mBio.01597-18 (PMC6199492; doi:10.1128/mBio.01597-18)
Supplement: TABLE S2 [file mbo005184118st2.docx]

**Table S2**. Cardiolipin species identification in *toxoplasma* by LC-HRMS.

| Species | m/z | Formula |
| --- | --- | --- |
| C68:5 | 1398.95658128 | C_77_H_140_O_17_P_2_ |
| C68:3 | 1402.98788144 | C_77_H_144_O_17_P_2_ |
| C70:5 | 1426.98788144 | C_79_H_144_O_17_P_2_ |
| C70:4 | 1429.00353152 | C_79_H_146_O_17_P_2_ |
| C72:7 | 1450.98788144 | C_81_H_144_O_17_P_2_ |
| C74:9 | 1474.98788144 | C_83_H_144_O_17_P_2_ |
| C74:8 | 1477.00353152 | C_83_H_146_O_17_P_2_ |
| C74:7 | 1479.01918160 | C_83_H_148_O_17_P_2_ |
| C74:5 | 1483.05048176 | C_83_H_152_O_17_P_2_ |
| C74:4 | 1485.06613184 | C_83_H_154_O_17_P_2_ |
| C74:3 | 1487.08178192 | C_83_H_156_O_17_P_2_ |
| C74:2 | 1489.09743200 | C_83_H_158_O_17_P_2_ |
| C74:1 | 1491.11308208 | C_83_H_160_O_17_P_2_ |
| C74:0 | 1493.12873216 | C_83_H_162_O_17_P_2_ |
| C76:9 | 1503.01918160 | C_85_H_148_O_17_P_2_ |
| C78:11 | 1527.01918160 | C_87_H_148_O_17_P_2_ |
| C78:9 | 1531.05048176 | C_87_H_152_O_17_P_2_ |
| C78:8 | 1533.06613184 | C_87_H_154_O_17_P_2_ |
| C78:7 | 1535.08178192 | C_87_H_156_O_17_P_2_ |
| C78:6 | 1537.09743200 | C_87_H_158_O_17_P_2_ |
| C78:5 | 1539.11308208 | C_87_H_160_O_17_P_2_ |
| C80:1 | 1575.20698256 | C_89_H_172_O_17_P_2_ |
| C82:11 | 1583.08178192 | C_91_H_156_O_17_P_2_ |
| C82:3 | 1599.20698256 | C_91_H_172_O_17_P_2_ |
| C82:0 | 1605.25393280 | C_91_H_178_O_17_P_2_ |

25 kinds of cardiolipin species were identified in HPLC-HRMS results. The first column showed the total number of carbons and unsaturation of each kind of cardiolipin, whose corresponding m/z is shown in the second column, while the formula of each kind of cardiolipin is described in the third column.
